# Overcoming Chemo-Mechanical Instability at Silicon-Solid Electrolyte Interfaces in Solid-State Batteries

**DOI:** 10.1021/acsami.5c11621

**Published:** 2025-10-21

**Authors:** Lammi Terefe Kitaba, Yosef Nikodimos, Semaw Kebede Merso, Bereket Woldegbreal Taklu, Gashahun Gobena Serbessa, Woldesenbet Bafe Dilebo, Tsung-I Yeh, Joshua Alexander Iskandar, Felika Valencia, Chia-Yu Chang, Chia Lung Hsieh, Shawn D. Lin, She-Huang Wu, Wei-Nien Su, Bing Joe Hwang

**Affiliations:** † Nano-Electrochemistry Laboratory, Department of Chemical Engineering, 34878 National Taiwan University of Science and Technology, Taipei 106, Taiwan; ‡ Nano-Electrochemistry Laboratory, Graduate Institute of Applied Science and Technology, 34878 National Taiwan University of Science and Technology, Taipei 106, Taiwan; § Sustainable Electrochemical Energy Development Center, 34878 National Taiwan University of Science and Technology, Taipei 106, Taiwan; ∥ National Synchrotron Radiation Research Center (NSRRC), Hsinchu 30076, Taiwan

**Keywords:** all-solid-state batteries, silicon anode, conductive agent, chemo-mechanics, in situ LiF formation

## Abstract

Silicon is the preferred choice for lithium-ion battery anodes due to its high theoretical capacity and low lithiation potential. However, achieving high areal capacity with silicon anodes in solid-state batteries (SSBs) is challenging because of poor electronic and ionic conductivity, as well as chemo-mechanical instability at the silicon|solid electrolyte (Si|SE) interfaces. Here, we propose fabricating and testing composite anodes made of nanosized Si powder embedded in partially fluorinated graphene (Si-FG) and Li_6_PS_5_Cl (LPSCl) sulfide SE. X-ray photoelectron spectroscopy revealed that the in situ formation of LiF-rich SEI can protect against SE decomposition at the interface in the Si-FG-LPSCl composite anode. FIB-SEM and EIS analyses also indicate a stable structure and low interfacial resistance after one cycle for a composite anode containing FG. The incorporation of partially FG enhances both electronic (through heterojunction formation with Si) and ionic conductivities, buffers significant volume changes, and ensures chemo-mechanical stability in the composite anode. The Si-FG-LPSCl composite anode in SSBs delivered high discharge/charge capacities of 3499/2994 mAh g^–1^ at a C-rate of C/20 and an ICE of 85.6% in a half cell. This work provides valuable insights for advancing high-capacity Si composite anodes to meet future energy needs.

## Graphical abstract

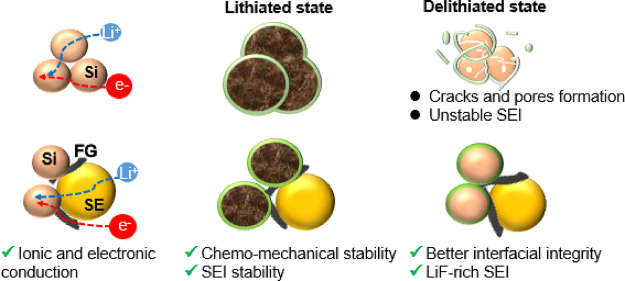

## 1 Introduction

It is essential to utilize high-capacity electrode materials to advance high-energy solid-state batteries (SSBs).^1^ The SSBs offer high energy density and enhanced safety due to nonflammability, thus claiming to be the next generation of energy storage devices.^2–6^ As a result of increased concern about CO_2_ emissions, it has driven the development of electric vehicles (EVs) as a substitute for the internal combustion engine,^2,7–10^ which calls for the advancement of SSBs with high energy density and improved safety; one of the key approaches is developing high-capacity anode materials.

Silicon (Si) is among the identified anode materials,^11–13^ having noticeable qualities of high specific capacity, 3579 mAh g^–1^ at room temperature, low working voltage (0.4 V vs Li^+^/Li), and is naturally abundant.^7,14,15^ However, Si adoption in SSBs faces a challenge due to volume change during cycling (about 300%),^16,17^ fast capacity decay, interface instability with solid electrolytes (SEs), chemo-mechanical instability, and solid electrolyte interface (SEI) degradation and growth.^18,19^ The low electronic and ionic conductivity intrinsic properties of silicon are also believed to have constrained the Si anodes’ rate performance.^20,21^

To address volume change limitation during charging/discharging, different Si structures such as nanoparticles,^22^ porous/hollow nanoparticles,^23,24^ and nanowires,^25^ have been utilized in SSBs with sulfide-based SEs due to exceptional ionic conductivity. Unlike in low-capacity materials, getting high performance in SSBs using high-capacity materials necessitates an increased local electron and Li^+^ ion flow near the active materials during the charge/discharge process.^21,26^ Recently, the potential of Si anodes in SSBs has been demonstrated through various manufacturing approaches, including thin-film fabrication,^27^ slurry preparation, and composite electrode development.^28^

Despite showing strong promise as an alternative to lithium metal anodes, these electrodes have either demonstrated high performance only at elevated temperatures or have been limited by the area loading of Si.^29^ This can be directly attributed to the electrode’s limited effective electronic and ionic conductivities. Si has an electrical conductivity of 10^–5^ S cm^–1^,^30,31^ and ion diffusivity in Si varies from 10^–16^ to 10^–8^ cm^2^ s^–1^.^32^

Furthermore, chemo-mechanical instability is a significant challenge in SSBs, resulting from the interaction between electrochemical reactions and mechanically induced stress and strain.^33^ Specifically, the chemo-mechanical behavior of Si composite anodes in SSBs is influenced by two significant challenges. (i) Si is unstable against sulfide SEs at low lithiation potentials, leading to SEI formation at the Si|SE interface and disturbing ion/electron percolation.^34^ (ii) The significant volume change of Si during lithiation/delithiation generates interfacial strains that mechanically damage the SE, causing cracks, contact loss, and impedance growth.^17,19^ These coupled electrochemical and mechanical degradations, ranging from electrode pulverization to interfacial fracture, are recognized as key factors limiting the performance and stability of SSBs.^35^

Current approaches to address these changes, as reported in the limited works, could be broadly classified into two categories. The first involves surface modification of the Si anodes through the application of protective coatings. Huo et al. introduced a thin polypropylene carbonate (PPC) layer on 2D Si sheets to suppress interfacial degradation and maintain stable contact.^34^ In contrast, Xu et al. introduced a LiAlO_2_ coating on Si particles to enhance ionic conductivity and mechanical strength simultaneously.^36^ Sun et al. designed a multifunctional SiO_2_-carbon coating on Si particles that provides efficient electron/ion transport and ensures mechanical stability during cycling.^37^ The second approach focuses on developing or tailoring SEs with improved stability. Huang et al. investigated hydride-based SEs, signifying that superior electrochemical stability with 3D Si anodes,^14^ and similarly, Han et al. demonstrated that employing a poly­(vinylidene fluoride-*co*-hexafluoropropylene) (PVDF-HFP)/LATP SE with a structurally modified Si by silver nanoparticle anode can effectively regulate electron/Li^+^ ion transport and SEI chemistry.^20^ Collectively, these studies with Si and SEs that provide interfacial stability against the Li metal anode,^38,39^ highlight the critical roles of both anode surface engineering and SEs design in achieving interfacial stability and high-performance in SSBs. Accordingly, a good interface for Si anodes should possess robust mechanical strength to withstand volume changes, maintain chemical stability to prevent unwanted reactions at the interface, and exhibit high conductivity to enhance the kinetics of Li^+^ ion diffusion. Incorporating Si into 2D structural carbon materials is crucial for addressing volume variation and low conductivity in Si electrodes.^40^ High-specific surface areas in these materials shorten electron transport and lithium ion diffusion paths, accommodate mechanical stress during Li-ion insertion and extraction, and prevent particle agglomeration.^41^ Hence, it is generally recommended that carbon and SEs can be added to buffer volume change and enhance electron and ion conduction in SSBs. However, sulfide-based SEs are unstable at the lithiated/Si interface, and carbon addition can speed up SEs decomposition.^34,42^

Herein, motivated by the idea of designing composite anodes (3D architecture), Si nanopowder embedded into partially fluorinated graphene (FG), hereafter Si-FG, and sulfide SE (Li_6_PS_5_Cl/LPSCl) are chosen to formulate the composite anode. Partially FG is a semiconductor with tunable band gaps, which can be adjusted by varying the fluorine content.^43–45^ Since covalent bonding alone reduces overall conductivity, the formation of heterojunctions, such as a p–n junction within partially FG, is recognizable (Figure S1, Supporting Note).^46^ Notably, when preparing composite materials from Si and other semiconductor materials, the contact between two semiconductors with different band gaps can form a heterojunction structure. This heterostructure creates a synergistic effect that enhances the charge transfer rate.^47,48^ Unlike most studies that focus on improving a single limitation of Si anodes, this work improves the performance of Si anodes from multiple perspectives. Incorporating partially FG into the Si-SE composite anode of SSBs offers several benefits. (1) Promotes chemo-mechanical stability at Si|SE interfaces by buffering huge volume changes of Si; (2) facilitates the formation of stable LiF-rich SEI between lithiated/Si and sulfide SE, which could be linked to the dissociation of fluorine from FG.^49^ (3) More importantly, it enhances electronic conductivity due to the heterojunction structure created between Si and partially FG; and (4) the tendency of Si particles agglomeration due to the high surface energy of nano-Si is significantly reduced.^50^

The X-ray photoelectron spectroscopy (XPS) and differential capacity (d*Q*/d*V*) analysis results indicated that no SE decomposition was observed. The promising Si-FG-LPSCl composite anode with sulfide-based SE in SSBs delivered high discharge/charge capacities 3499/2994 mAh g^–1^ (corresponding to areal capacity 7.35/6.28 mAh cm^–2^) at a C-rate of C/20, and an initial Coulombic efficiency (ICE) of 85.6% in a half cell.

## 2 Experimental Section

### 2.1 Silicon-Fluorinated Graphene Composite Preparation

The Si-FG composite was prepared through a simple hydrothermal process. In a typical process, Si powder (nanocrystalline, <150 nm, 99% purity, from Strem Chemicals, Inc.), ball milled at 500 rpm for 5 h, and FG (53% F content, 4–10 μm, from Macklin) by a ratio of 92:8, respectively, were added into anhydride ethyl alcohol solvent. The suspension was first subjected to ultrasonication for 30 min. It was then transferred to a high-pressure autoclave with Teflon for further treatment. This process was conducted at 190 °C for 12 h in an autoclave and then allowed to cool naturally to room temperature. The precipitates were collected by centrifugation, washed with ethyl alcohol five times, and vacuum-dried at 70 °C for 12 h to obtain the Si-FG composite material.

### 2.2 Composite Anode Preparation

As prepared, the Si-FG composite and SE (LPSCl, NEI Corporation) were mixed using a mortar and pestle. A 3:2 weight ratio of Si-FG and LPSCl was used to fabricate the Si-FG-LPSCl composite anode. FG with high fluorine content is used as a conductive additive, comprising 8 wt % of the Si-FG composite and 4.8 wt % of the Si-FG/LPSCl (3:2) composite anode. The experiments were conducted within a glovebox filled with argon (Ar) gas, ensuring that the levels of oxygen and water were kept below 0.1 ppm.

The composite cathode preparation for assembling SSBs was produced by mixing lithium niobate-coated single-crystal NMC811 (Nb@S-NMC811, ALEEES, Taiwan), used as received, LPSCl, and VGCF in a mass ratio of 75:23:2, respectively, using a mortar and pestle for 30 min.

### 2.3 Material Characterization

The SEM and EDX on FE-SEM (Field Emission Scanning Electron Microscopy, JSM 6500F, FE-SEM, JEOL) and Focused Ion Beam (FIB-SEM, FEI Helios 600i) were used to characterize the structural and morphological evolution of the Si-containing anodes before and after lithiation/delithiation. The elemental compositions and distributions were analyzed by energy-dispersive X-ray spectroscopy (EDS). The transmission electron microscopy (TEM) instrument (FEI TecnaiTM G2 F-20 S-TWIN) running at 200 kV was used to perform TEM and high-resolution TEM (HRTEM) analyses. The XPS was conducted at the beamline station BL 24Al. At the National Synchrotron Radiation Research Center (NSRRC), Hsinchu, Taiwan. The energy calibration of the entire XPS spectrum was performed using the Au 4f_7/2_ peak at 84.1 eV as a reference. The X-ray diffraction (XRD) was measured on a Bruker D2 phaser-diffractometer with copper (Cu Kα) radiation in the range of 10–90 (2θ angle). The sample was pelletized, and the powder was used to measure the XRD. The sample was sealed in an XRD holder to protect it from air exposure. The Raman spectra were analyzed using a Uni-RAM spectrometer with a 532 nm laser beam, with a silicon wafer serving as a reference. The samples were measured under a sealed transparent glass sample holder.

### 2.4 Electrochemical Test and Cell Assembly

The SSBs were assembled by the powder pressing method in the glovebox. To construct the half-cells, 100 mg of LPSCl SE was first pressed in a Teflon die of 10 mm diameter under a pressure of 125 MPa. Subsequently, different amounts of anodes: 1.656 mg of Si, 2.76 mg of Si-LPSCl composite anode containing 60 wt % active material Si (with Si mass of 1.656 mg), and 3 mg of Si-FG-LPSCl composite containing 1.8 mg Si-FG (with Si mass fixed at 1.656 mg) were cast on SE and pressed to 380 MPa. The counter electrode was prepared using an In–Li alloy, after which the entire cell was further pressed at 100 MPa to ensure good interfacial contact between the components. The mass of Si was used as a basis to calculate specific capacity. The full-cell test was fabricated in the same way as the half-cell, except that the In–Li was replaced with an NMC811 composite cathode, which was pressed together with the anode at 380 MPa. Stainless steel (SUS) was used as the current collector on both sides for the half-cell and full-cell. Finally, the cell remains intact by tailor-made cell (TS cell) framework under a torque application of 7 N m. SSBs were conducted in a battery tester (Arbin, BT-2000). The charge and discharge tests were performed at room temperature, with a voltage range of −0.6 to 0.9 V (vs In–Li) for the half-cell and 2.4–4.2 V for the full-cell.

Electrochemical impedance spectroscopy (EIS) was conducted using a potentiostat (Metrohm, Autolab), and the measurements were taken in the frequency range of 10 MHz to 10 mHz. The AC amplitude was 10 mV. The data was analyzed and evaluated using RelaxIS software. The electronic conductivity of the three anodes was determined through DC polarization measurement using an ion-blocking setup on a potentiostat (Biologic, VMP-300). A mass of 30 mg was placed in a die, sealed, and compressed under a uniaxial pressure of 3.5 tons for 2 min. The cell voltage of composite anodes was systematically varied between −25 and 40 mV. For Si anode, the voltage is from 30 to 45 mV in 5 mV increments. Each applied voltage was held for 3 h to stabilize the measured electronic current, followed by a 15 min rest between consecutive steps. Cyclic voltammetry was performed on a potentiostat (Biologic, VMP-300) in the voltage range of −0.6 to 0.9 V (vs In–Li) at a scan rate of 0.1 mV s^–1^.

## 3 Results and Discussion

### 3.1 Characterization of Si-FG Composite

The composite of Si-FG was prepared through a simple self-assembly process, as illustrated in Figure S2. Consequently, the Si nanopowder crystalline structures and the synthesized Si-FG composite were characterized by XRD in Figure 1a to evaluate structural changes. The XRD spectra display the distinctive peaks of Si nanopowder at 33.2°, 55.5°, 66.2°, and 82.4° in both samples, corresponding to the cubic Si planes (111), (220), (311), and (400).^51,52^ This indicates that the crystallinity of Si in the Si-FG composite remains unchanged. However, the zoom in around peak (111) has been found to shift slightly toward the lower 2θ value (Figure 1b). This suggests that the variation in the lattice parameter of the crystalline structures could result from phase separation of the Si and SiO_*x*_ oxide layer.^53^ This result aligns with HRTEM images showing an increase in lattice spacing of Si in the Si-FG composite, indicating a structural change in crystalline Si, as illustrated in the enlarged image in Figure 2h.

**Figure 1:**
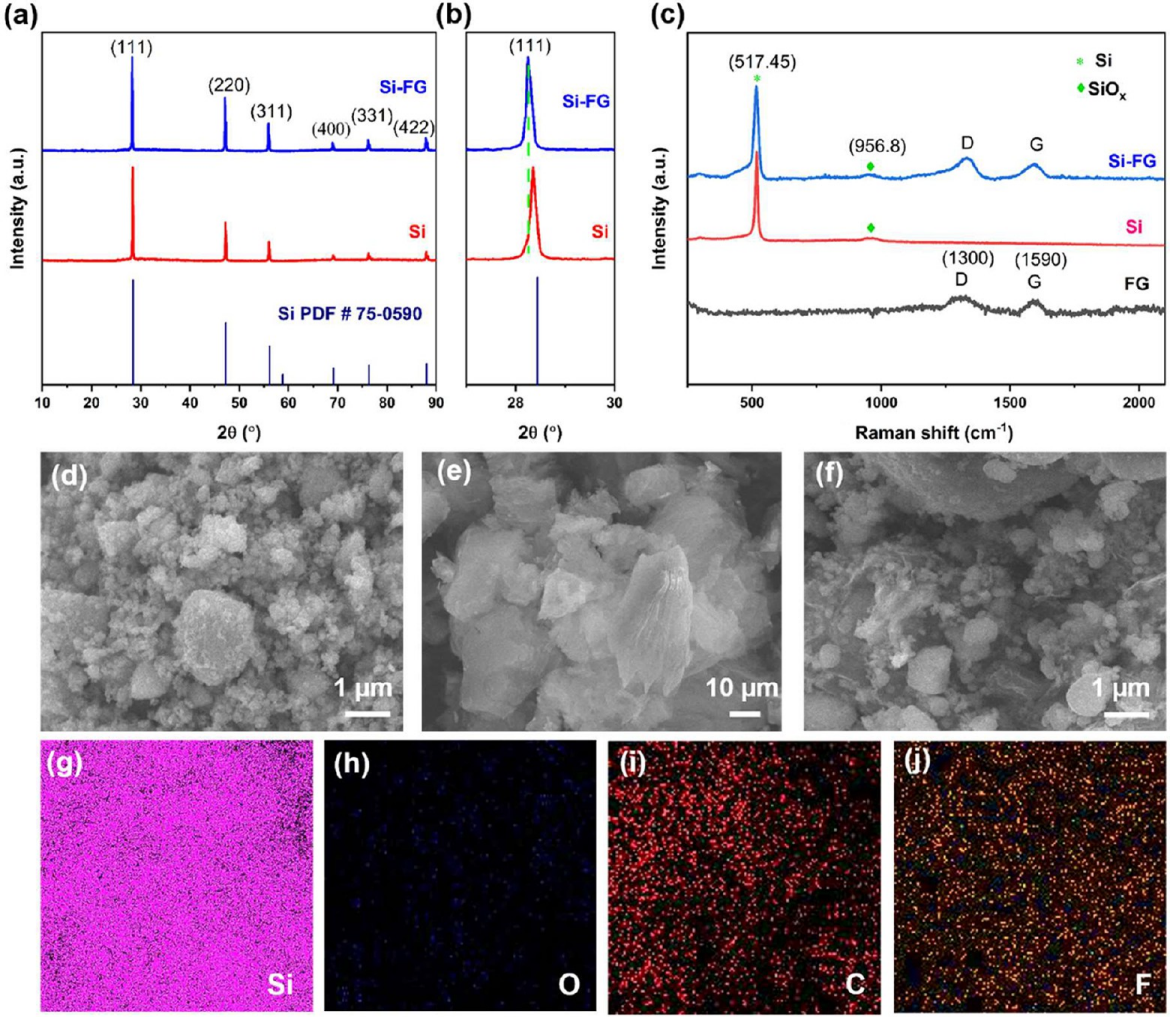
Characterization of Si-FG composite: XRD patterns of Si and Si-FG composite (a), zoom in around peak (111) (b), Raman spectrum of FG, Si, and Si-FG composite (c). Surface SEM images of Si (d), FG (e), Si-FG (f), and EDS elemental mapping of Si-FG: Si (g), O (h), C (i), and F (j).

**Figure 2:**
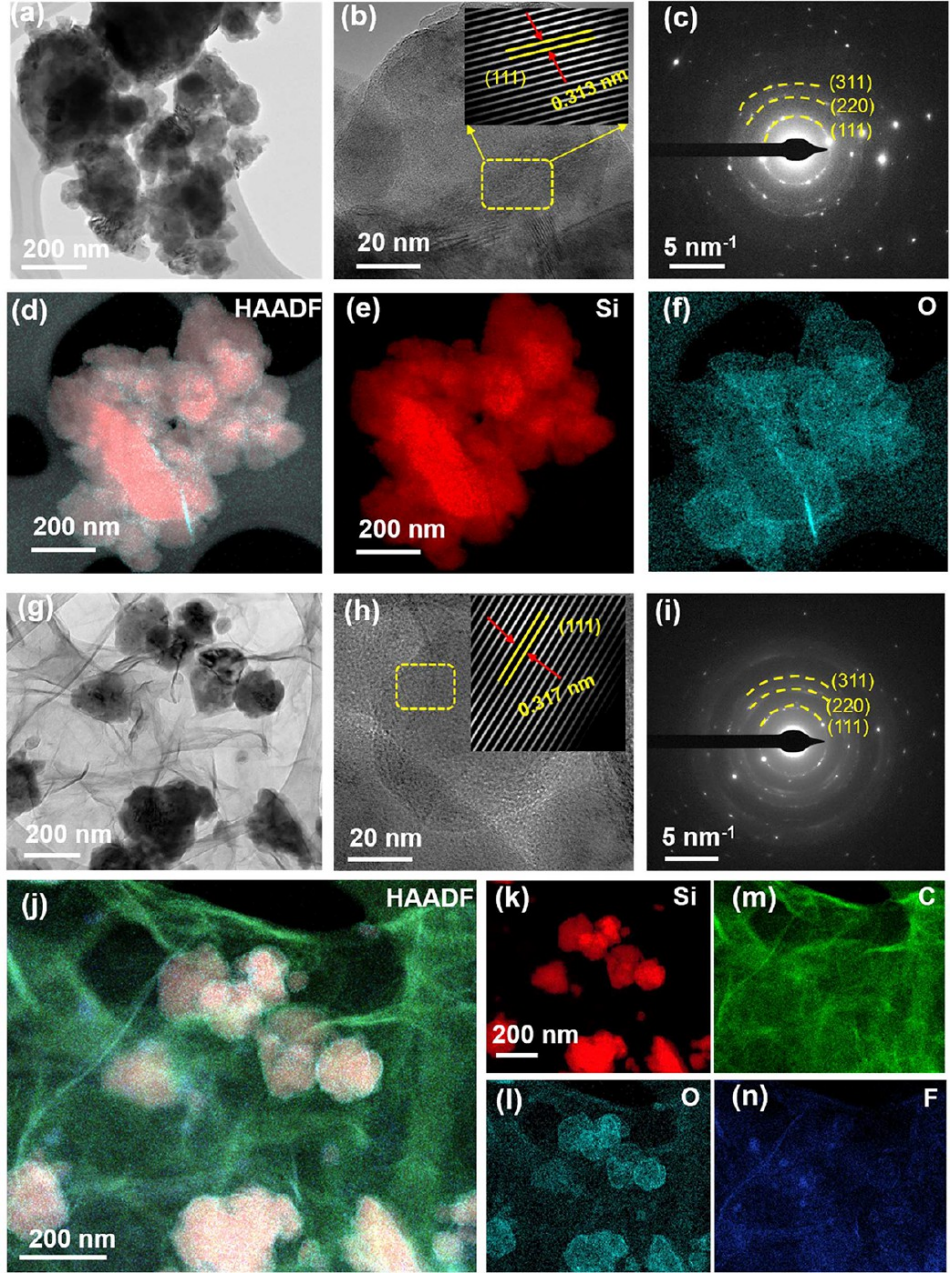
TEM image (a), HRTEM and inset enlarged images (b), SAED pattern (c), HAADF image (d), and corresponding EDS mapping (e, f) of Si. TEM image (g), HRTEM and inset enlarged images (h), SAED pattern (i), HAADF image (j), and corresponding EDS mapping (k–n) of Si-FG composite.

The Raman spectroscopy was conducted to provide insight into the fundamental characteristics of FG. A characteristic D peak at ∼1300 cm^–1^ due to disorder and a G peak at ∼1590 cm^–1^ belong to pristine FG. The peak intensity ratio of the D to G peak (*I*_D_/*I*_G_) of pristine FG and Si-FG after the formation of the composite was assessed and found to be the same (*I*_D_/*I*_G_ ≈ 1.3), as shown in Figure S3a. This is due to the defluorination of FG, which can resist thermal reduction below 300–400 °C. The 2D band at approximately 2850 cm^–1^ decreases and broadens as the fluorination rate increases, and is suppressed in the region highlighted after 50% fluorination, indicating high structural disorder (Figure S3b). After 50% fluorine coverage, the 2D Raman signal is nearly quenched, suggesting minimal residual sp^2^ carbon.^54^

The Raman spectra of the Si-FG composite exhibit a distinct peak at 517.45 cm^–1^, corresponding to the Si component. In addition, two prominent peaks are visible at ∼1300 and 1590 cm^–1^. These are the D (disorder) and G (graphitic) carbon bands in FG. The broad peak at 956.8 cm^–1^ shows an amorphous layer of SO_*x*_ (oxide of Si) (Figure 1c).^52^ Overall, the vibrational modes in the Raman spectra of Si for both samples remain unchanged, indicating that the Si does not chemically interact with FG.

The SEM images of Si, FG, and Si-FG composite materials are presented in Figure 1d–f, respectively. The EDS elemental mapping of the Si-FG composite (Figure 1g–j) and evenly distributed elements demonstrates the successful preparation of the composite material.

TEM and HRTEM were further employed to examine the microstructure of the Si-FG composite material. Figure 2a shows a TEM image of Si, while Figure 2b presents the HRTEM image of Si, which reveals a lattice fringe spacing of 0.313 nm corresponding to the (111) plane, along with an enlarged inset image. Figure 2c depicts the selected area electron diffraction (SAED) pattern of Si, Figure 2d displays a high-angle annular dark-field (HAADF) image of Si, and Figure 2e,f illustrate elemental EDS mapping of Si, highlighting the presence of an oxide layer. Conversely, Figure 2g is a TEM image of the Si-FG composite, and Figure 2h, along with the inset enlargement, shows HRTEM images of the Si-FG composite exhibiting the (111) plane of Si within the Si-FG composite. The measured lattice fringe is 0.317 nm, and the enlarged inset image indicates the expansion of crystalline Si due to phase separation with the SiO_*x*_ oxide layer during hydrothermal processing. This result is consistent with the XRD diffraction pattern of the two samples (Figure 1b). Figure 2i presents the SAED pattern of Si-FG, while Figures 2j and S4 are the HAADF images of the Si-FG composite, revealing that Si nanoparticles are embedded into the FG sheets, indicating that Si nanoparticle agglomeration is considerably inhibited. Figure 2k–n provides the corresponding elemental EDS mapping of the Si-FG composite.

### 3.2 Morphological and Interfacial Evolution of Si-FG-LPSCl Composite Anode

The morphological change and structural stability of silicon can impact battery performance, as Si undergoes volume changes during charge and discharge.^2^ This morphological appearance of the electrodes was examined using SEM before cycling (Figure S5) and FIB-SEM along with FIB cross-sectional elemental mapping (Figures S6–S8) at pristine, after lithiation, and delithiation states in a half-cell. The magnified cross-sectional views of these FIB-SEM are presented in Figure 3. SEM images of pristine electrodes before the battery test are presented in Figure S5a–c. After applying 380 MPa of high pressure during fabrication, FIB-SEM revealed that the silicon nanoparticles in the Si anode become aggregated, densified, and contain pores (Figure 3a). In contrast, when the Si-LPSCl anode is pressed to 380 MPa, the relatively soft LPSCl endorses close contact with Si particles, resulting in the formation of pores between Si particles (Figure 3b). For the Si-FG-LPSCl anode, pressing to 380 MPa led to a more integrated structure and fewer small pores (Figure 3c). During the lithiation process, lithium-Si alloys (Li_*x*_Si) form, leading to an increase in volume. The Si anode transforms into a uniform amorphous structure,^55^ and the electrode remains intact without developing cracks or pores (Figure 3d). In comparison, in the Si-LPSCl anode, even if the interface between Si and LPSCl remains integral, SE bulk cracks are observed in Figure 3e. This induces stress and strain to be generated in the cell due to Si’s huge volume expansion,^34^ suggesting that the utilization of Si composite anodes needs to overcome the chemo-mechanical effect. Here, the LPSCl’s confinement helps reduce pore formation, as the interface between LPSCl and Si-LPSCl remains in close contact. At the interface between SE and Si, where the growth of the interphase is persistent and tends to be uneven under high current density, the projection of the interphase generates significant localized stress. The development of stress and strain in the cell leads to cracks and fractures in the SE, ultimately causing the composite anode cell to fail. In the case of Si-FG-LPSCl, the anode consists of clusters between components with small interparticulate spaces, but no bulk SE crack was observed. Therefore, it is evident that incorporating FG can alleviate the chemo-mechanical effect in the Si composite anode, and the layered structure effectively buffers the volume changes of Si. The large clusters are likely composed of the Li_*x*_Si alloy (Figure 3f). As a result, combining Si with the FG and LPSCl enhances the stability of the electrode structure by reducing the generation of LPSCl bulk cracks, thereby ensuring effective charge transfer connections. After the first delithiation, extracting Li from Li_*x*_Si back to Si triggers a significant volume contraction, which challenges the structural stability of the Si electrodes. In the Si anode, numerous new pores and cracks are generated (Figure 3g). The generated pores result from the volume shrinkage that occurs during the delithiation process. These pores and cracks can hinder the conduction of ions and electrons, resulting in slow kinetics and a decrease in capacity.^56^ In the Si-LPSCl composite anode, submicro cracks are observed due to the shrinkage of the Si volume (Figure 3h). In contrast, Si-FG-LPSCl exhibits the most stable structure (Figure 3i). Si–FG–LPSCl displays a denser and more uniform morphology after first delithiation than Si and Si–LPSCl anodes. This occurs because FG and LPSCl particles are dispersed within the Li_*x*_Si aggregations, promoting a constant delithiation process and preventing the development of pores. In general, the captivity provided by FG and LPSCl helps minimize Si aggregation during lithiation and reduces pore formation during delithiation. As a result, combining Si with the FG and LPSCl enhances the stability of the electrode structure by reducing pores and cracks, thereby ensuring effective charge transfer connections.

**Figure 3:**
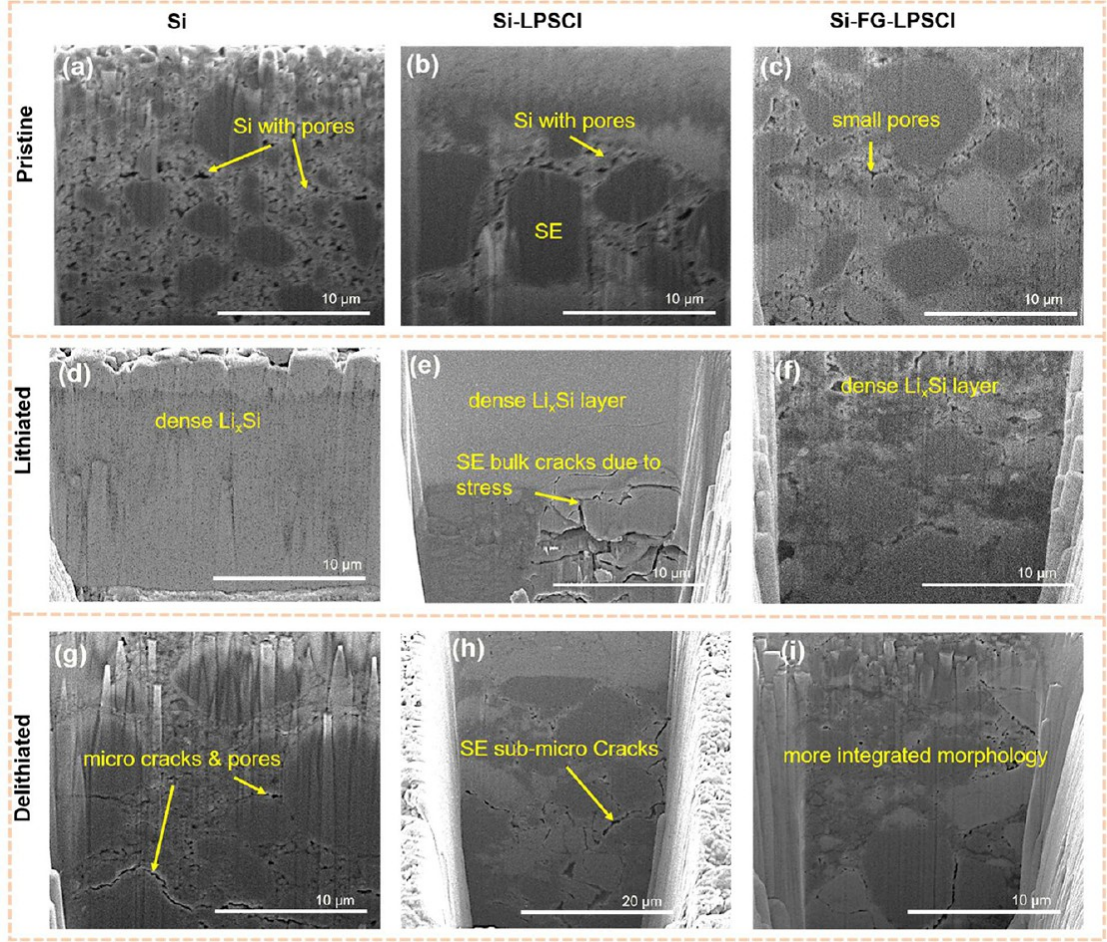
Morphology of Si anodes evolution analysis: Cross-sectional FIB-SEM of pristine state Si (a), Si-LPSCl (b), and Si-FG-LPSCl (c); lithiated Si (d), Si-LPSCl (e), and Si-FG-LPSCl (f), delithiated Si (g), Si-LPSCl (h), and Si-FG-LPSCl (i), in half-cell configuration.

XPS was used to assess the (electro-)­chemical stability of sulfide-based SE with the two Si composite anodes in cell configurations: Si-LPSCl||In–Li and Si-FG-LPSCl||In–Li. After applying a current density of 0.5 mA cm^–2^ for one cycle, the electrochemical degradation species at the Si|SE and Si-FG|SE interfaces were investigated.

The Si 2p XPS spectra, pristine (before cycling) and after initial delithiation of the Si-LPSCl composite anode, are shown in Figure 4a. In the pristine state, a noticeable peak indicating Si is observed at around 98.8 eV, along with a peak corresponding to SiO_*x*_ at around 103.1 eV.^34^ This suggests the presence of an impurity, specifically an oxidized layer, on the Si powder surface. There is also the possibility of further oxidation during the preparation of the Si-FG composite. After one cycle, there is a significant reduction in the Si peak, accompanied by a new peak corresponding to SiO_2_ at 102.9 eV and Li_*x*_SiO_*y*_ at 101.1 eV due to the reaction between lithium and SiO_*x*_. This has been put forward by Huo et al.^34^ that the disproportionation of SiO_*x*_, described by the reaction: SiOx=(x/2)SiO2+(1−x2)Si is influenced by the lithiation process of the resulting Si, leading to the formation of SiO_2_ along with a Li_*x*_SiO_*y*_ phase.

**Figure 4:**
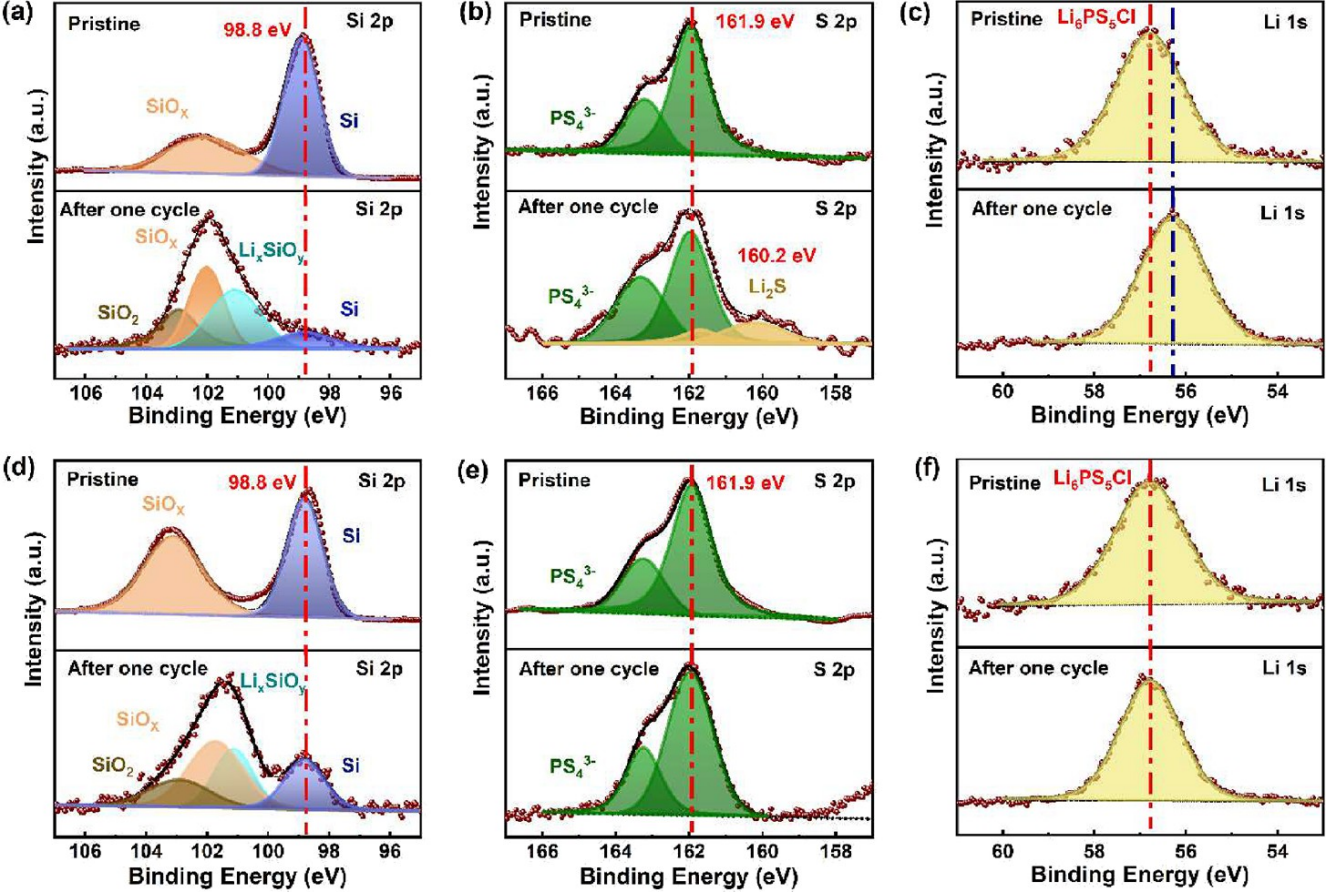
Electrochemical stability analysis of Si-SE and Si-FG-SE composite anodes: XPS spectra of Si 2p (a), S 2p (b), and Li 1s (c) for Si-LPSCl anode, and Si 2p (d), S 2p (e), and Li 1s (f) for Si-FG-LPSCl anode, at pristine state (top) and after initial delithiation (bottom).

The S 2p XPS spectra, both pristine and after initial delithiation of the Si-LPSCl anode, are shown in Figure 4b. In the pristine state, double peaks are increased at 161.9 eV in the S 2p spectra, attributed to PS_4_^3–^ in the argyrodite LPSCl structure.^42^ After one cycle, the double peaks at 160.2 eV in the S 2p spectra suggest the formation of Li_2_S, originating from the reaction of LPSCl with lithium. The decomposition of LPSCl contributes to the continuous growth of the SEI. Despite the deconvolution of the Li 1s spectra (Figure 4c) being challenging due to various Li^+^ species, noticeable shifts to lower binding energies indicate a reduction of Li^+^ from the original SE.^42,57^

Figure 4d shows Si 2p spectra for the Si-FG-LPSCl composite anode in both pristine and after one cycle. In pristine, peaks at 98.8 and 102.0 eV are observed, corresponding to Si and SiO_*x*_, respectively. After one cycle, similar to the behavior observed in the Si-LPSCl composite anode as seen in Figure 4a, the reaction between SiO_*x*_ and lithium results in the formation of phases at 102.72 eV (SiO_2_) and 101.1 eV (Li_*x*_SiO_*y*_).

Figure 4e presents the XPS spectra of S 2p for the Si-FG-LPSCl composite anode in both pristine and after one cycle. Pristine, the peaks at 161.9 eV correspond to PS_4_^3–^ a unit of the LPSCl. After one cycle, there was no observed peak at around 160.1 eV in the S 2p spectra, indicating (electro-)­chemical stability at the interface of the Si-FG composite and SE due to the LiF-rich SEI formed during lithiation. In addition, the F 1s spectra (Figure S9a) reveal a peak at 685.47 eV after one cycle, confirming the formation of LiF in the SEI.^58–60^ From this spectral analysis, both before and after cycling, the persistence of semi-ionic and covalent C–F bonds is also observed, along with the formation of LiF, confirming that FG remains at the interface and continuously contributes to interfacial stabilization. In the C 1s spectra, the C–C (284.8 eV) and C–CF (286.5 eV) peaks are retained, C–F (288.7 eV), and C–F_*x*_ (290.5 eV) bonding are still evident after cycling (Figure S9b),^61^ indicating that FG remains chemically stable to a significant extent and continues to influence the Si-electrolyte interface. This confirms the persistent beneficial effect of FG modification of the Si anode. From Figure S9c, the spectra of Cl 2p show no observable change of Cl ions between the pristine and after the first cycle. There is no observed shift of peaks before and after one cycle (Figure 4f), despite the challenge of deconvolution in the spectra of Li 1s. Among the various Li^+^ containing species, LiF formation can stabilize SEI. It shows that SiO_*x*_, an impurity at the silicon powder surface, can contribute to the SEI creation in both Si-LPSCl and Si-FG-LPSCl composite anodes.

### 3.3 Electrochemical Measurements

The cyclic voltammetry was analyzed for the Si-FG-LPSCl composite anode to assess the electrochemical stability in more detail during lithiation and delithiation. In–Li is used as a counter electrode. The reduction peak for the Si-FG-LPSCl electrode was observed starting from 0.27 V, which is a typical lithiation feature of Si,^2^ and the oxidation potential started at 0.32 and 0.52 V, which is delithiation characteristic of Si,^62,63^ showed stable cycling for subsequent five cycles without showing SE decomposition in the first lithiation and delithiation (Figure 5a). In all three electrodes, there is also no other potential plateau (reduction peaks) during lithiation, and no extra oxidation peaks during delithiation were observed apart from the one corresponding to Si (Figures 5b and S10a,b). SE decomposition is negligible, even for a Si-LPSCl composite anode. Further, the cyclic voltammetry was analyzed for the FG-LPSCl anode, and it was confirmed that the FG did not induce SE decomposition. During the first lithiation, a reduction peak above 1.5 V (vs Li^+^/Li), further confirming the formation of a LiF-rich layer,^64^ and the reduction peak at 1.5 V during the cathodic scan signifies the formation of the SEI layer,^65^ which is no longer observed in the following cycles, suggesting that the SEI remained stable. From the cyclic voltammetry test, unlike other conductive carbon materials, there was no decomposition of SE in the voltage window of the battery test, as shown in Figure S11.

**Figure 5:**
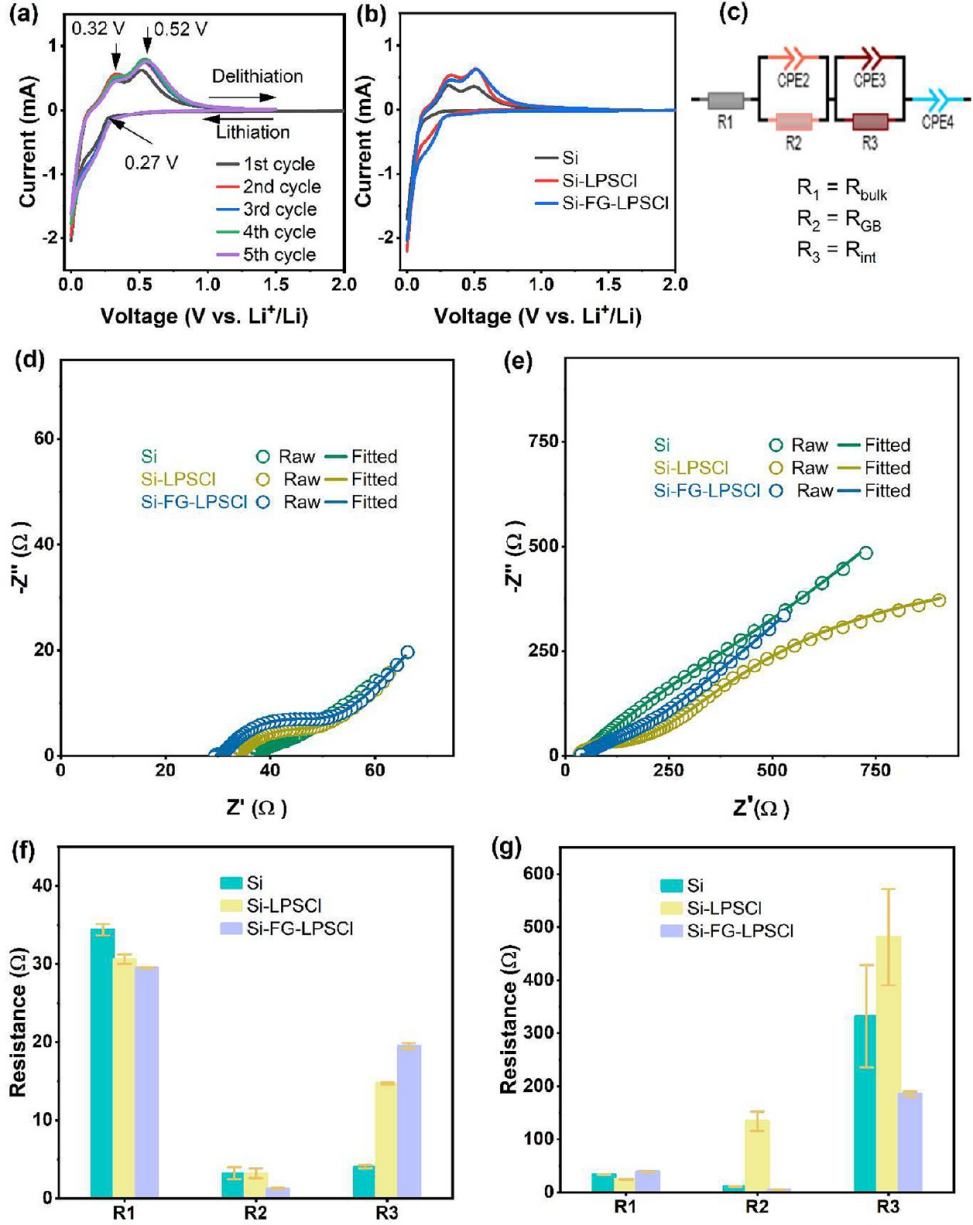
CV profile of Si-FG-LPSCl anode (a), the first CV profile of Si anodes: Si, Si-LPSCl, and Si-FG-LPSCl (b). Equivalent circuit fitting diagram (c), the Nyquist plots of Si anodes after the first discharge (lithiation) (d) and after the first charge (delithiation) (e); the corresponding fitted parameters result after lithiation (f) and delithiation (g).

EIS was employed to assess the stability of Si||In–Li, Si-LPSCl||In–Li, and Si-FG-LPSCl||In–Li cells after first lithiation and delithiation at 0.5 mA cm^–2^ current density, as shown in Figure S12. Before the battery testing, Nyquist plots exhibited incomplete semicircles in all three cells, followed by Warburg (diffusion) tails (Figure S13). The total (bulk) resistance is at the point of interception. The Si–FG–LPSCl anode exhibits the lowest bulk resistance because of the combination of FG (a higher electron conductor compared to Si) and LPSCl (a higher ion conductor compared to Si), which reveals the importance of electron and ion conduction in electrode materials. On the other hand, the Si-LPSCl configuration exhibits a higher bulk resistance than pure Si. This is because Si has higher electrical conductivity than LPSCl.

Further to quantify the effective electronic conductivity of the Si-FG-LPSCl composite anode, the ion-blocking cell was assembled and assessed through DC polarization for the three anodes, as shown in Figure S14a–f. The Si anode exhibited 8.2 × 10^–5^ S cm^–1^. The Si-LPSCl anode exhibited a low electrical conductivity of 3.6 × 10^–5^ S cm^–1^. On the other hand, the Si-FG-LPSCl anode exhibited the highest electrical conductivity of 8.4 × 10^–3^ S cm^–1^. From this DC polarization test, Si-FG-LPSCl exhibits about 102 times the effective electronic conductivity of Si.

The equivalent circuit (EC) used to represent the impedances of the three anodes after lithiation and delithiation states, along with circuit elements: *R*_1_ – (*P*_2_)­(*R*_2_) – (*P*_3_)­(*R*_3_) – *P*_4_, where *R* (resistance) and *P* (constant phase element/CPE) are shown in Figure 5c. After lithiation, the Nyquist plots at high and mid frequencies exhibit a depressed semicircle, whereas at low frequencies, they display Warburg diffusion tails (Figure 5d). The midfrequency semicircle is attributed to the combined resistances of interfaces (*R*_int_) at the SE|Si anodes and SE|In–Li,^2^ denoted by *R*_3_. Since cold-pressed cells frequently include grain boundaries (GB), the depressed semicircle at a high frequency is thought to represent the grain boundary resistance (*R*_GB_), represented by *R*_2_. The bulk resistance (*R*_bulk_) in the cells is represented by *R*_1_. After one cycle, small noticeable semicircles appear in the Nyquist plots, unlike in the lithiation states, and a lengthy tail appears in the three cells’ configuration (Figure 5e). The resistance values of the three cells after lithiation are presented in Figure 5f, and Table S1 summarizes the EC element values. From the fitted result, Si exhibits the lowest resistance at the interfaces (4.07 Ω), Si-LPSCl demonstrates a higher resistance (14.73 Ω), and Si-FG-LPSCl indicates the highest resistance (19.52 Ω).

The delithiation state was examined using the same analogous circuit, and the results are shown in Figure 5g, and Table S2 provides details of the corresponding circuit elements’ values. *R*_int_ evolution of Si, Si-LPSCl, and Si-FG-LPSCl anodes containing cells was 331.93, 481.13, and 185.00 Ω, respectively. The increase in *R*_int_ (*R*_3_) observed in all cells during full delithiation can be attributed to both the structural and electrochemical changes of the Si anodes. Li‘s presence within Si improves interfacial contact and conductivity. However, upon full delithiation of Si, the complete extraction of Li leads to significant volume contraction and loss of conductive pathways. Effect of these factors on the interfacial impedance after delithiation also reported by Cao et al.^1^ These results show considerably increased *R*_int_ in cells with Si and Si-LPSCl configurations. The significant increase in *R*_int_ is due to structural and chemo-mechanical instability. Conversely, the low *R*_int_ observed in Si-FG-LPSCl results from its improved charge transfer rate, stable structure, and chemo-mechanical stability. The composite electrodes consist of numerous grains of active material and SE particles, across which Li^+^ ions must migrate. Under ideal conditions, ion transport occurs continuously through these GBs.^66–68^ However, chemo-mechanical stress during cycling can generate fractures, interface cracks, and even particle pulverization. Then, it disrupts the continuity of Li^+^ pathways, forcing ions to detour or tunnel across voids, which in turn increases the resistance at GBs. This aspect is well unveiled by the distribution of relaxation times (DRT) curves (Figure S15c,d). Incorporating FG mitigates this effect by providing a flexible and conductive framework that buffers Si expansion, maintains particle contact, and suppresses the growth of GBs resistance during cycling.

Furthermore, DRT analysis was used, which allows for the identification of time scales and the reliable separation of contributions from interface (SEI and charge transfer) resistances. Therefore, the electrochemical response can be decoupled into distinct contributions: bulk resistance (*R*_bulk_) and grain boundary resistance (*R*_Gb_) (above ∼10^–6^ s), SEI resistance (*R*_SEI_, ∼10^–6^–10^–3^ s), charge transfer resistance (*R*_ct_, ∼10^–2^–1 s), and diffusion resistance (*R*_diff_, ∼1–10 s).^69,70^

After lithiation (Figure S15a,b) in the *R*_SEI_ region, the Si-FG-LPSCl anode shows a noticeably shorter relaxation time constant, reflecting faster interfacial kinetics. At the same time, the peak intensities remain comparable, suggesting that FG incorporation improves Li^+^ transport efficiency due to LiF-rich SEI phase formation. In contrast, Si-LPSC shows the longest relaxation time due to the formation of more resistive decomposition products (3D interface) (Li_2_S, LiCl, and Li_3_P) in SEI.^71^ In the region *R*_ct_, although the relaxation time (τ) across Si, Si-LPSCl, and Si-FG-LPSCl anodes remains unchanged, the Si-FG-LPSCl exhibits the largest DRT peak intensity, indicating that LiF-rich surface increases the charge-transfer resistance without altering the fundamental kinetics. In essence, the LiF-rich SEI stabilizes the interface both mechanically and chemically, being ionically conductive and electronically insulating.^72^ It does not slow the reaction itself but rather imposes an additional resistive layer at the interface, which is in good agreement with the EC results shown in Figure 5f.

After the first cycle (Figure S15c,d), the Si-LPSCl shows the most significant increase in *R*_SEI_ and *R*_ct_ peaks, indicating stress-induced instability that leads to SEI degradation and interfacial cracking, thereby impeding charge transport. For the Si-FG-LPSCl anode, the *R*_SEI_ and *R*_ct_ remain low after one cycle, demonstrating that the incorporation of FG stabilizes the Si|LPSCl interface, suppresses chemo-mechanical degradation, and preserves fast interfacial kinetics. DRT curves clearly show that the Si-FG-LPSCl exhibits the lowest *R*_int_ (*R*_SEI_ + *R*_ct_), confirming its more stable interfacial behavior compared to Si and Si-LPSCl anodes after cycling, which is in good agreement with EC values (Figure 5g). It is important to note that structural stability, driven by chemo-mechanical resilience, is crucial for Si-SE composite anodes, especially during the initial lithiation process.

Figure 6a shows the preparation and cell assembly of the Si-FG-LPSCl composite anode. The charge/discharge performance of these electrodes with sulfide-based SE was examined in a half-cell with In–Li serving as the counter electrode. The active mass loading of Si in each anode was 2.1 mg cm^–2^ at the rate of C/20 (1 C = 3500 mA g^–1^), which corresponds to 0.58 mA cm^–2^ current density, and in the 0–1.5 V voltage window (vs Li^+^/Li) at room temperature. The galvanostatic charge–discharge curve of the Si anode during the first three cycles is depicted in Figure 6b. The Si delivered the lowest 3360/1889 mAh g^–1^ discharge–charge specific capacities, respectively, and the lowest ICE of 56.2%. Figure 6c displays that the Si-LPSCl anode provided larger discharge and charge specific capacities of 3408/2793 mAh g^–1^, respectively, and an ICE of 81.95%. In comparison, Si-FG-LPSCl anode delivered the largest 3499/2994 mAh g^–1^ (corresponding to the areal capacity of 7.35/6.28 mAh cm^–2^), discharge–charge specific capacities, respectively, and high ICE of 85.6% (Figure 6d).

**Figure 6:**
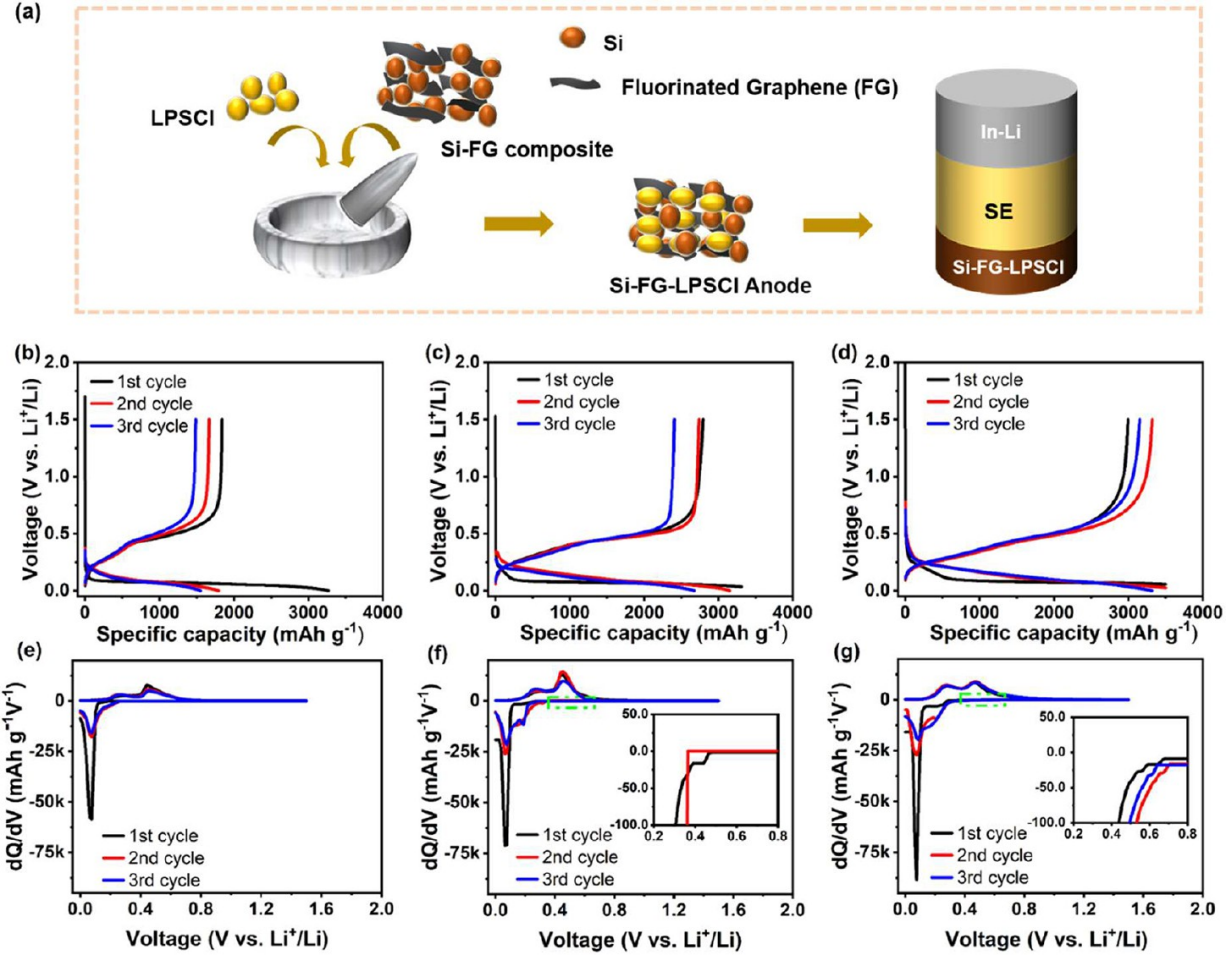
Si anodes electrochemical performance in half-cell configuration in Si|SE|In–Li. Si-FG-LPSCl composite anode preparation and cell assembly illustration (a). For the charge/discharge curve: Si (b), Si-LPSCl (c), and Si-FG-LPSCl (d), the half-cell assembly procedure is the same as described in (a). The corresponding d*Q*/d*V* of Si anodes: Si (e), Si-LPSCl (f), and Si-FG-LPSCl (g).

The differential capacity (d*Q*/d*V*) curve of the three Si anodes was plotted to assess the electrochemical stability of the Si-SE composite anode. The corresponding differential capacity curve of the Si anode is shown in Figure 6e. The plot shows more pronounced capacity fading for the Si anode, indicated by the decreasing area below the curve, which is associated with the initial capabilities for lithiation and delithiation.^73^ From the d*Q*/d*V* of Si-LPSCl anode, during the initial discharge, a noticeable peak is observed at around 0.06 V, possessing a 0.12 V onset potential (Figure 6f). This route denotes the formation of metastable amorphous Li_*x*_Si by a reaction of solid-state amorphization.^74^ Before discharging to 0.25 V, no noticeable peaks are seen, implying a slight decomposition of SE at the interface.^1^ The result agrees with the XPS spectra around the S 2p and Li 1s regions (Figure 3b,c). The voltage drops rapidly to under 0.3 V vs Li^+^/Li during the first lithiation. The small, tiny voltage inclination between 0.26 and 0.11 V vs Li^+^/Li and extended voltage plateau up to the 0 V cutoff voltage. It can be observed that a slight slope between 0.26 and 0.11 V vs Li^+^/Li is affected by the irreversible lithiation of silicon oxide surfaces.^14,75^ Two prominent oxidation peaks at around 0.26 and 0.47 V are seen in the subsequent dealloying process and are attributed to the phase transition from Li_3.17_Si to Li_7_Si_3_ and subsequently to LiSi.^76^ Conversely, during the second discharge phase, two peaks at 0.22 and 0.01 V represent the opposite conversions from LiSi to Li_7_Si_3_ and finally to Li_3.17_Si.^76^ The following charging phase does not have an oxidation peak that resembles the first cycle, which indicates chemo-mechanical instability.

In comparison, the d*Q*/d*V* profile of Si-FG-LPSCl (Figure 6g) demonstrates that no peaks have been seen before 0.27 V (vs Li^+^/Li). A peak at 0.11 V vs Li^+^/Li denotes a long plateau at the initial lithiation. The charge/discharge is more comparable to those found in liquid electrolytes.^77^ Like the Si-LPSCl anode, there is a slight inclination between 0.27–0.11 V vs Li^+^/Li, which is prejudiced by the irreversible lithiation of surface impurity of the Si (silicon oxide) layer. The voltage plateau signifies the crystalline Si lithiation during the initial dealloying. The discharge curve from the second cycle exhibits a different shape, marked by a voltage slope starting at approximately 0.31 V, indicating the amorphous Si state of lithiation. Therefore, the highest specific capacities and ICE in Si-FG-LPSCl anode suggest that compositing Si-FG and SE could enhance Si utilization in SSBs. The galvanostatic charge–discharge curve of the Si-FG-LPSCl composite anode for higher Si loadings was evaluated, as shown in Figure S15, further demonstrating the reliability of the Si-FG-LPSCl composite anode. A Si active mass loading of 4.2 mg cm^–2^ at a C-rate of C/20 (corresponding to 1.15 mA cm^–2^ current density) delivers an initial discharge/charge capacity of 1138.9/935.6 mAh g^–1^, with an ICE of 82.1% (Figure S16a). Similarly, a higher mass loading of 6.3 mg cm^–2^ at a C-rate of C/20 (corresponding to 1.73 mA cm^–2^ current density) achieves the initial discharge/charge capacities of 1135.2/922.5 mAh g^–1^, with an ICE of 81.2% (Figure S16b). In addition, the Si-FG composite with different FG loadings (5 and 10 wt %) was tested. In comparison, 5 wt % discharge/charge 3499.6/2887.3 mAh g^–1^ with an ICE of 82.5%, while 10 wt % delivered 3498.7/2747.9 mAh g^–1^ with an ICE of 78.5%, due to excessive FG reducing active Si utilization (Figure S17a,b). Thus, 8 wt % FG (4.8 wt % in Si-Fg-LPSCl) was identified as the optimal loading, offering the best electrochemical performance.

Overall, the synergistic effect that enhances the charge transfer rate is attributed to heterojunction formation in the Si-FG composite, and FG can buffer volume changes to accommodate the stress and strain generated during charge and discharge. Then, it enhances cell performance. Table S3 presents a summary of the key literature comparisons, showing how the Si-FG-LPSCl composite anode addresses the typical initial discharge and charge limitations of silicon anodes.

The XRD patterns of the Si-FG-LPSCl composite anode in its pristine state and after the first electrochemical cycle were obtained (Figure S18). A noticeable decrease in the Si diffraction peak intensity is observed after cycling, reflecting the progressive amorphization of crystalline Si nanoparticles. These nanoparticles undergo structural disordering upon cycling, leading to an amorphous phase that suggests the efficient utilization of the active Si within the composite anode.^1^

Before cycling, the Raman spectra of the Si-FG-LPSCl composite show the PS_4_^3–^ peak at 424 cm^–1^,^71^ Si peak at 517.45 cm^–1^, along with the D (1300 cm^–1^) and G (1590 cm^–1^) bands of FG, confirming the presence of graphitic domains.^54^ A broad peak at 956.8 cm^–1^ is also observed, indicating a surface SiO_*x*_ layer.^52^ After cycling, these vibrational features remain essentially unchanged. The D and G bands persist without noticeable degradation. This consistency demonstrates that FG retains structural stability and continues to provide a conducive, protective role (Figure S19).

As the cathode is also key in outlining the performance of a full cell, the In–Li is used in a half-cell to measure the performance of the NMC811 (Nb@S-NMC811) composite cathode in NMC811|SE|In–Li, cell configuration. Figure S20a presents the composite cathode charge/discharge profiles at the rate of C/20 for the first three cycles evaluated. For 1 C = 200 mA g^–1^, based on the mass of the NMC811 (active material). The test was conducted in the 2.5 and 4.3 V (vs Li^+^/Li). The charge and discharge capacity obtained at the first cycle were 195.9 and 135.5 mAh g^–1^, respectively, with an ICE of 69.2%. The half-cell charge–discharge capacities and d*Q*/d*V* of the NMC cathode in Figure S20b indicate that the NMC811 composite cathode exhibits irreversibility and some instability in its performance.

To demonstrate the outstanding electrochemical stability and the potential of the Si-FG composite material in SSBs, a full cell was constructed using a Si-FG-LPSCl composite anode, an LPSCl SE, and an NMC811 composite cathode. LPSCl was used in the composite cathode and anode formulation. The negative to positive (N/P) ratio is set to 1.37, based on the discharge capacities of Si in the half-cell (3000 mAh g^–1^) and NMC811 (200 mAh g^–1^).

Figure 7a shows the charge and discharge profile of Si-FG-LPSCl||NMC811 full-cell configuration. The initial charge and discharge capacities were 184.95/126.94 mAh g^–1^ (equivalent to 4.24 and 2.91 mAh cm^–2^), respectively, with an ICE of 68.6%. Figure 7b compares the cycling performance of the three anodes in full cells at a C-rate of C/10 with the first two cycles measured at C/20. From the first two cycles at a C-rate of C/20, the specific discharge capacities of Si are 104.9 and 99.2 mAh g^–1^ (corresponding to areal capacities of 2.41 and 2.28 mAh cm^–2^). In comparison, the Si-LPSCl anode exhibits 112.2 and 107.9 mAh g^–1^ (corresponding to areal capacities of 2.58 and 2.48 mAh cm^–2^). On the other hand, the Si-FG-LPSCl anode exhibits 132.5 and 133.5 mAh g^–1^(corresponding to areal capacities of 3.05 and 3.07 mAh cm^–2^). This result also provides strong evidence that incorporating FG into the Si-LPSCl composite can effectively address issues that hinder the initial utilization of silicon. In addition, these values are presented in areal capacity, as shown in Figure S21, for the full-cell SSBs tests. At the first C/10 C-rates, Si anode demonstrates an areal capacity of 1.52 mAh cm^–2^, Si-LPSCl shows 1.89 mAh cm^–2^, and Si-FG-LPSCl achieves 2.20 mAh cm^–2^ with capacity retention of 36, 9, and 40% after 50 cycles, respectively. Although the Coulombic efficiency of the NMC811 cathode used has shown irreversibility and some instability, the full cell results using Si-FG-LPSCl composite anode demonstrate good electrochemical stability and less polarization compared to Si-LPSCl and Si anodes. Besides the cycle stability, rapid increases in the average Coulombic efficiency are observed over the subsequent cycles. An average CE of 97.06% in Si-FG-LPSCl, 94% in Si-LPSCl, and 96% in Si can be achieved over 50 cycles. Figure S22 compares the stability of Si and Si-FG-LPSCl anodes. Si exhibits instability after 62 cycles, even though the Si-FG-LPSCl shows some capacity fading due to various factors; however, stability is achieved up to 74 cycles. Here, the cycle stability of Si is higher than that of the Si-LPSCl composite anode. This difference can be attributed to the chemo-mechanical instability effect in the Si-LPSCl anode.

**Figure 7:**
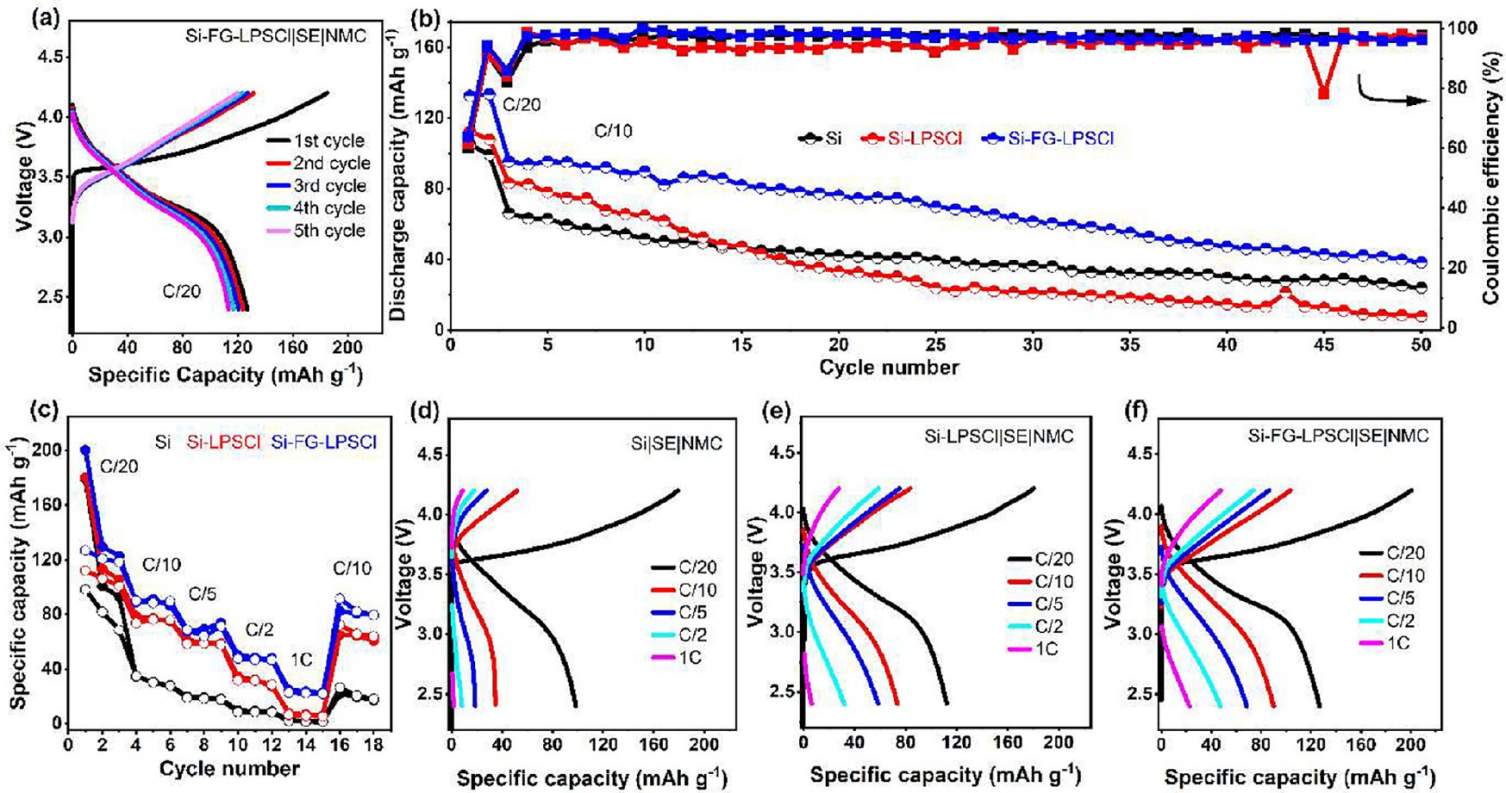
Electrochemical performance of Si anodes in a full-cell. The full-cell performance of SiFG-LPSCl at C/20 (a). Cycling performance comparison of Si anodes in full cell with high loading of cathode, 22.93 mg cm^–2^ at C/10 (b); rate capability of the three Si anodes (c), charge–discharge profiles of the three Si anodes at different C-rates: Si (d), Si-LPSCl (e), and Si-FG-LPSCl (f).

The electrochemical rate capability of Si, Si-LPSCl, and Si-FG-LPSCl anodes in full cells using the NMN811 composite cathode was evaluated. As shown in Figure 7c, the Si anode cell exhibited the lowest average discharge capacities of 83, 31, 18, 8, and 1.5 mAh g^–1^ when cycled at C/20, C/10, C/5, C/2, and 1C, respectively. On the other hand, the Si-LPSCl cell, when cycled at C/20, C/10, C/5, C/2, and 1C, delivers lower average capacities of 106, 75, 58, 31, and 6 mAh g^–1^, respectively. By contrast, the Si-FG-LPSCl anode cell shows the highest average discharge capacities of 122, 89, 68, 47, and 22 mAh g^–1^ when cycled at C/20, C/10, C/5, and 1C, respectively. This reveals that adding LPSCl and FG can enhance the electrochemical performance of the silicon anode due to improved charge transfer kinetics and chemo-mechanical stability between the Si-FG composite and LPSCl at the interface. The three Si anodes charge–discharge curves at different C-rates show that the Si-FG-LPSCl composite anode exhibited the best stability (Figure 7d–f).

## 4 Conclusions

This work provides valuable insights into the utilization of FG to achieve high capacity in Si anode paired with a lithium argyrodite sulfide SE (LPSCl), at room temperature. We systematically examined the electrochemical stability and chemo-mechanics at the interface between Si|LPSCl and Si-FG|LPSCl through XPS, FIB-SEM, and EIS analysis. More specifically, no apparent decomposition of SE was observed in the composite containing FG. Incorporating FG reduced mechanical stress and cracking caused by the severe volume change of the Si anode during charge/discharge, ensuring that the Si-FG|SE interface remains intact. As a result, cracks and fractures at Si-FG|SE interfaces and in the bulk SE were reduced, leading to a decrease in interfacial resistance.

In addition, the enhanced charge transfer kinetics and chemo-mechanical stability enabled the Si–FG–LPSCl anode configuration to achieve the highest Si utilization, with a discharge/charge capacity of 3499/2994 mAh g^–1^ at C/20. An ICE of 85.6% was achieved in the half-cell. From the full cell electrochemical performance, the Si-FG-LPSCl anode provides a high discharge capacity of 127 mAh g^–1^ at C/20 and exhibits better cycling stability. These values are significantly higher than those of the Si-LPSCl composite and Si anodes. These findings highlight the positive impacts of partially FG incorporated into the Si-SE composite anode on structural stability, interfacial resistance, and cell performance in SSBs.

FG can mitigate chemo-mechanical degradation in the composite Si-SE anode of SSBs. The addition of partially FG enables a robust Si composite anode with rapid effective conductivity of ions and electrons for next-generation SSBs applications.

## Supplementary Material



## Notes

FG structure and Si–FG composite synthesis; structural, spectroscopic (Raman, XRD, XPS), and microscopic (HAADF, SEM, FIB-SEM/EDS) analyses; electrochemical measurements (CV, EIS, DRT, charge–discharge, and conductivity); and performance comparison of Si anodes in half-cell and full-cell SSBs (PDF)
