# Supplementary material for: Overcoming Chemo-Mechanical Instability at Silicon-Solid Electrolyte Interfaces in Solid-State Batteries
Source: ACS Appl Mater Interfaces. 2025 Oct 21;17(44):60411–25. doi: 10.1021/acsami.5c11621 (PMC12598704; doi:10.1021/acsami.5c11621)
Supplement: Supplementary file 1 [file am5c11621_si_001.pdf]

Supporting Information

Overcoming Chemo-Mechanical Instability at Silicon-Solid Electrolyte Interfaces in Solid-State Batteries

Lammi Terefe Kitaba [a], Yosef Nikodimos [a,c], Semaw Kebede Merso [a], Bereket Woldegbreal Taklu [a], Gashahun Gobena Serbessa [a], Woldesenbet Bafe Dilebo [a], Tsung-I Yeh [a], Joshua Alexander Iskandar [a], Felika Valencia [a], Chia-Yu Chang [b], Chia Lung Hsieh [a], Shawn D. Lin [a,*], She-Huang Wu [b,c,*], Wei-Nien Su [b,c,*], Bing Joe Hwang [a,c,d,*]

[a] Nano-electrochemistry Laboratory, Department of Chemical Engineering, National Taiwan University of Science and Technology, Taipei 106, Taiwan

[b] Nano-electrochemistry Laboratory, Graduate Institute of Applied Science and Technology, National Taiwan University of Science and Technology, Taipei 106, Taiwan

[c] Sustainable Energy Development Center, National Taiwan University of Science and Technology, Taipei 106, Taiwan

[d] National Synchrotron Radiation Research Center (NSRRC), Hsinchu 30076, Taiwan

[*]Corresponding authors:

Email:

sdlin@mail.ntus.edu.tw (S. D. Lin)

wush@mail.ntust.edu.tw (S.-H. Wu)

wsu@mail.ntust.edu.tw (W.-N. Su)

bjh@mail.ntust.edu.tw (B. J. Hwang)

SUPPORTING NOTE

In fluorinated graphene (FG), the introduced fluorine atoms alter the hybridization of carbon atoms from sp^2 to sp^3, disrupting the conjugated π bond, as shown in Figure S1. This SP^3 hybridization also shifts the charge density and modifies the scattering centers within the conduction band. The extremely high electronegativity of fluorine atoms further localizes the electrons. This contributes to the widening of the band gap. As a result, with an increasing F/C ratio, FG gradually transitions from a conductor to a semiconductor and eventually to an insulator.[1, 2]

Compared to existing studies that have focused on structurally and electronically characterizing FG with defined stoichiometry, such as fully fluorinated (CF_1), partially FG sheets (CF_x, where x < 1) remain less experimentally explored, especially in terms of their C-F bonding configurations, electrical properties, and thermal stabilities.[3]

Partially FG, especially with about 50% fluorine coverage, can show different electronic behaviors based on how the fluorine atoms are arranged. For example, less stable setups, such as those with fluorine on only one side, can be either semiconducting or metallic. But the more stable setup, with fluorine on both sides, is expected to be metallic with no band gap at 50% coverage.[4] Altering the fluorine content of partially FG allows for the adjustment of its tunable band gaps. Since covalent bonding alone leads to a reduction in overall conductivity, the formation of heterojunctions, such as a p-n junction within partially FG, is recognizable.[3]

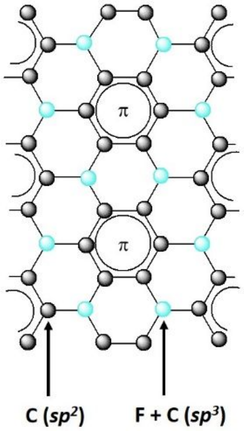

Figure S1. Scheme to illustrate the structure and electronic conduction properties of partially fluorinated graphene

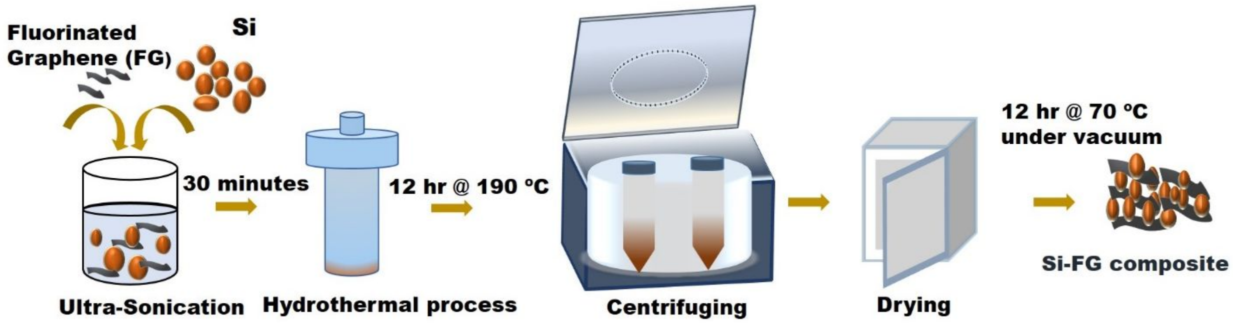

Figure S2. Schematic description of the synthesis process of Si-FG composite via the hydrothermal method.

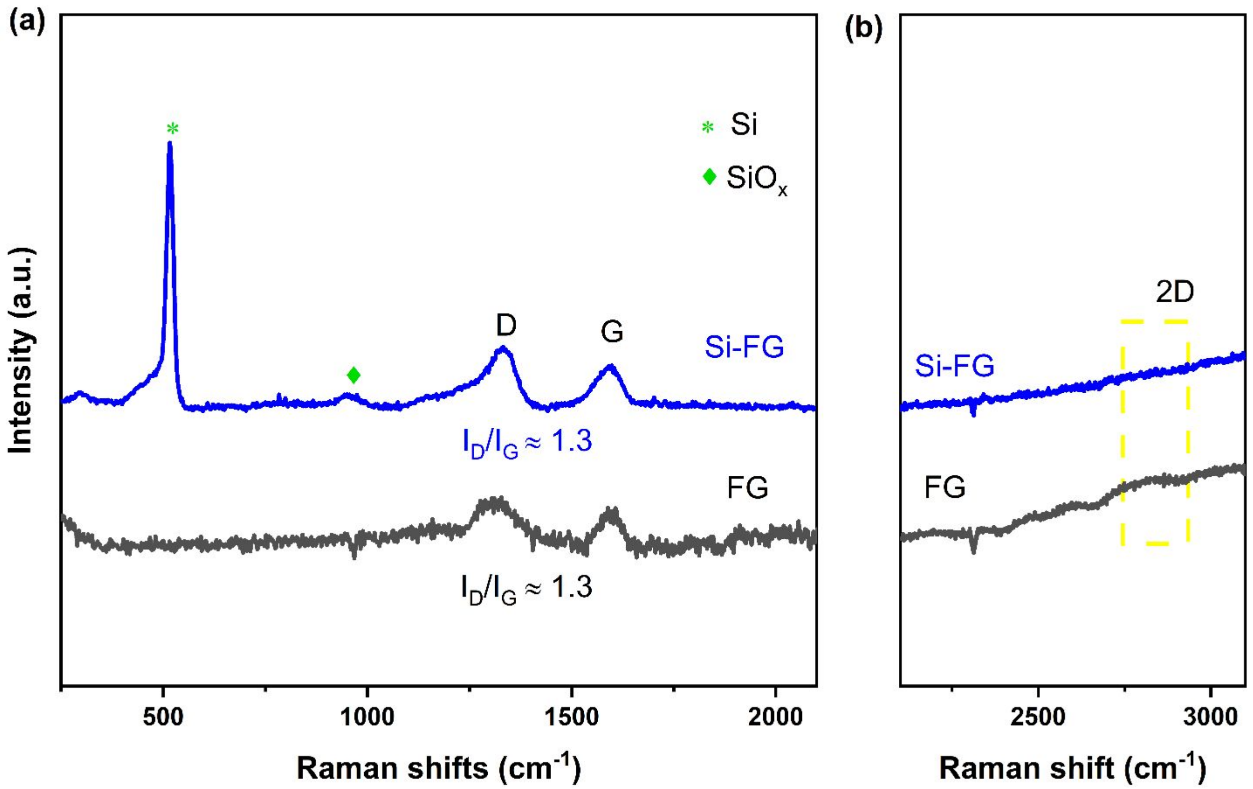

Figure S3. Raman spectra of FG and Si-FG composite: analysis of the D to G peak intensity ratio (I_D/I_G) (a), and 2D band indicating high structural disorder (b).

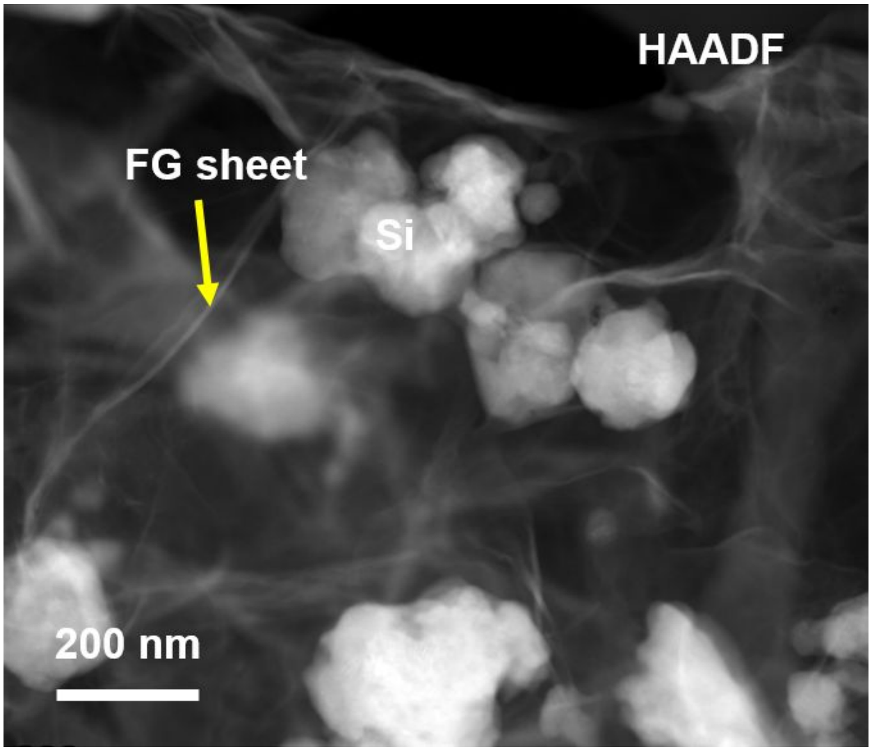

Figure S4. High-angle annular-field (HAADF) image of Si-FG composite

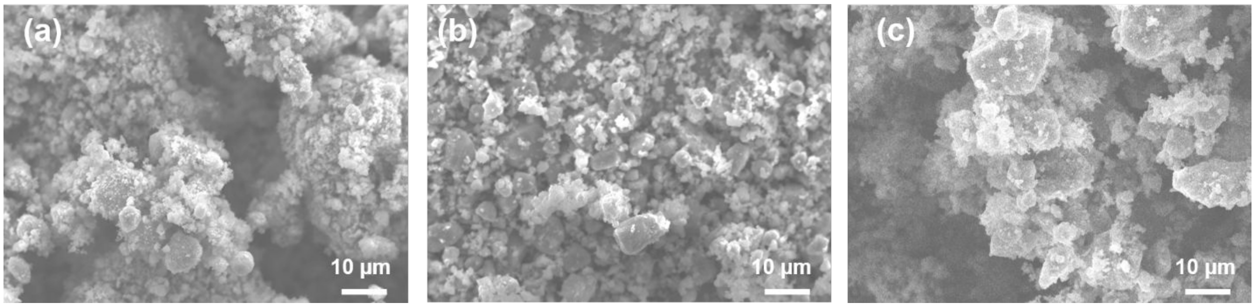

Figure S5. SEM Images of Si powder (a), Si-LPSCl composite (b), and Si-FG-LPSCl composite (c).

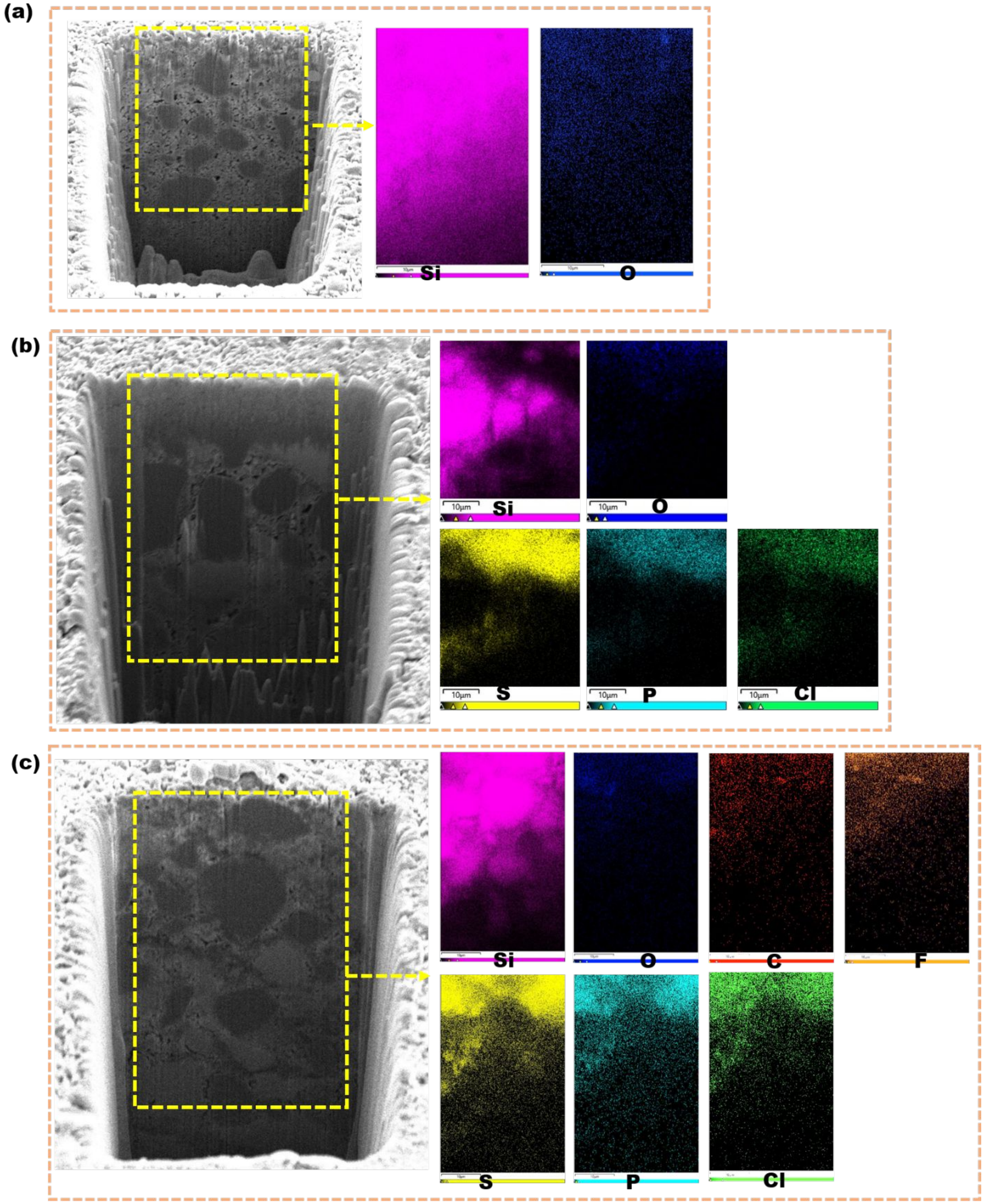

Figure S6. FIB cross-sectional elemental mapping of pristine electrodes after application of 350 MPa, Si (a), Si-LPSCl (b) and Si-FG-LPSCl (c)

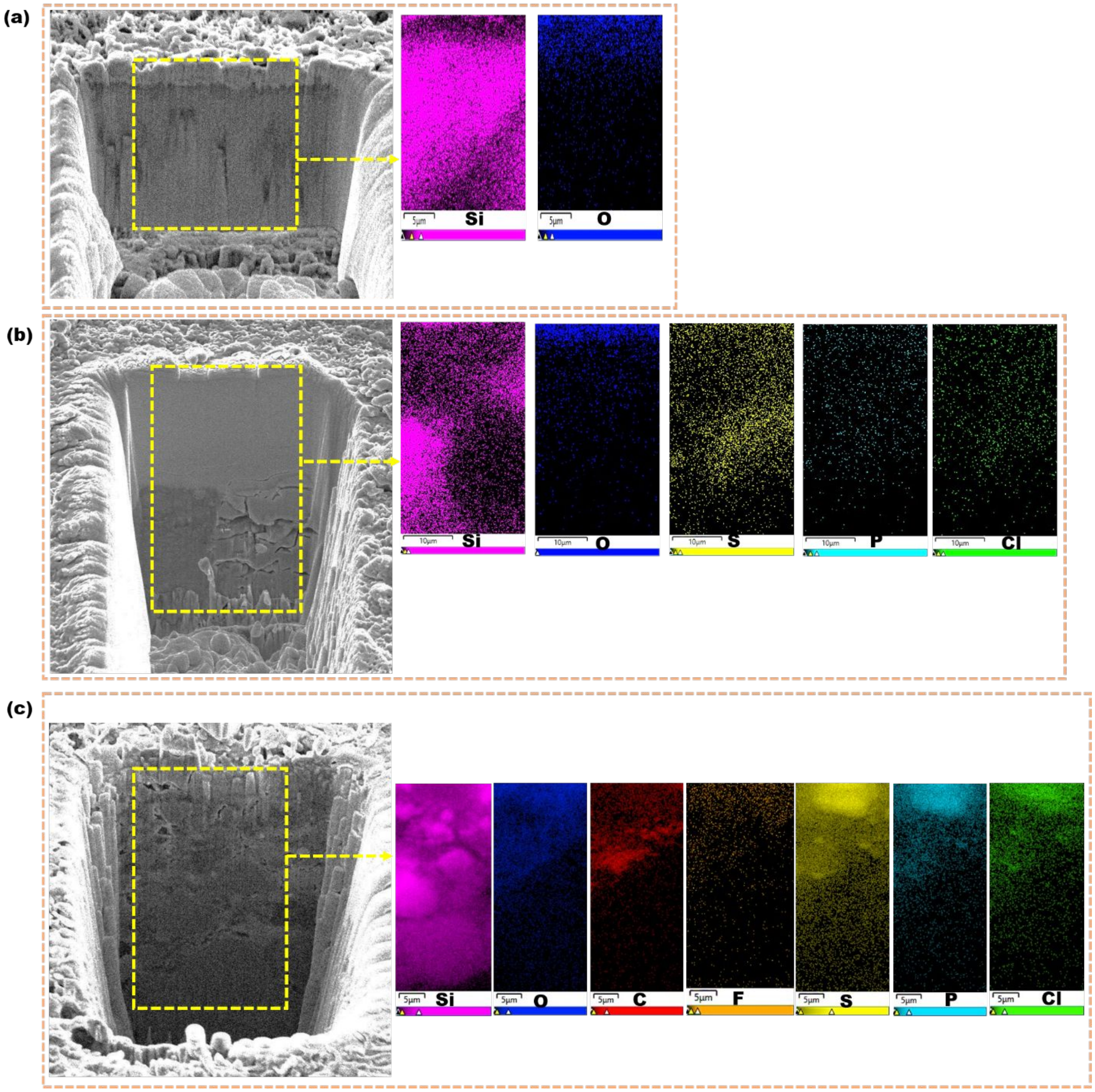

Figure S7. FIB cross-sectional elemental mapping of the electrodes after lithiation: Si (a), Si-LPSCl (b) and Si-FG-LPSCl (c).

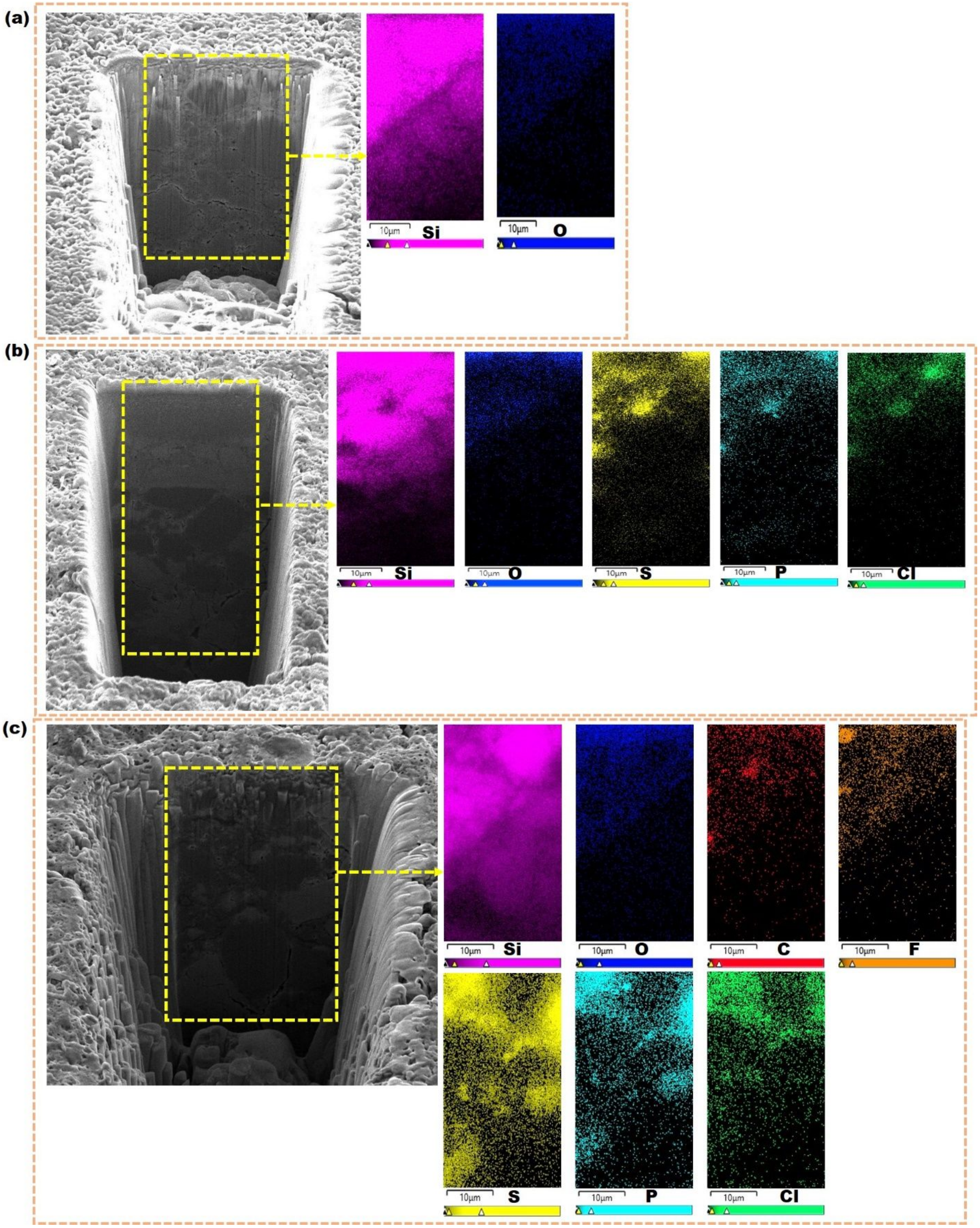

Figure S8. FIB cross-sectional elemental mapping of the electrodes after delithiation: Si (a), Si-LPSCl (b) and Si-FG-LPSCl (c).

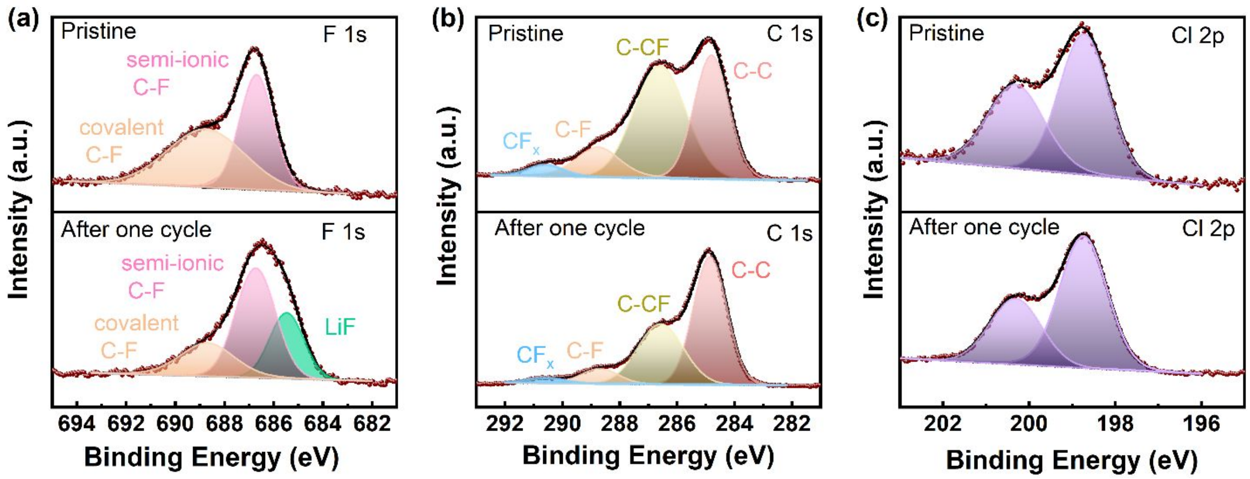

Figure S9. XPS spectra of F 1s (a), C 1s (b), and Cl 2p (c) for Si-FG-LPSCl composite anode in the pristine (top) and after one cycle (bottom).

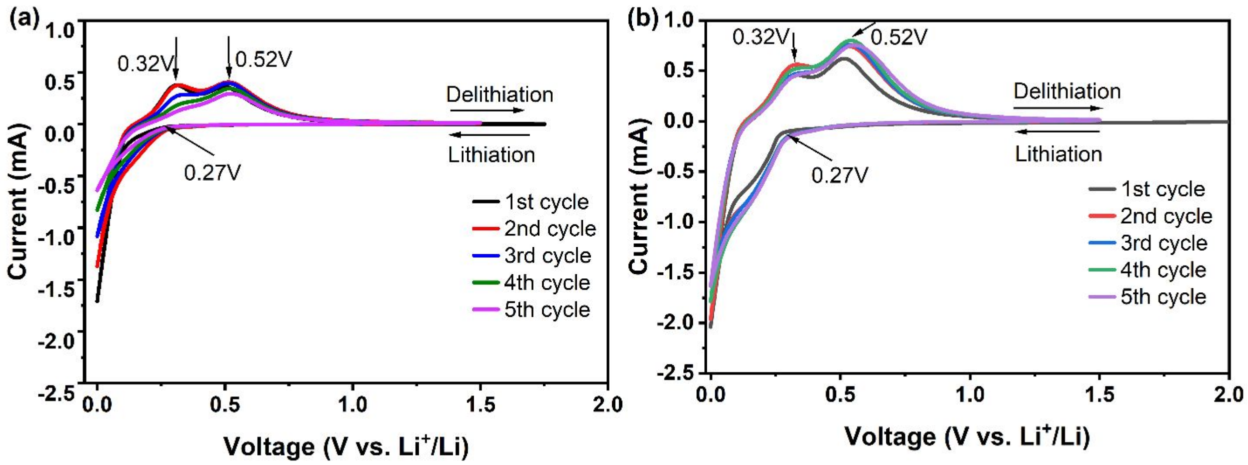

Figure S10. Typical CV profile of Si anodes in the first five cycles, Si (a) and Si-LPSCl composite (b).

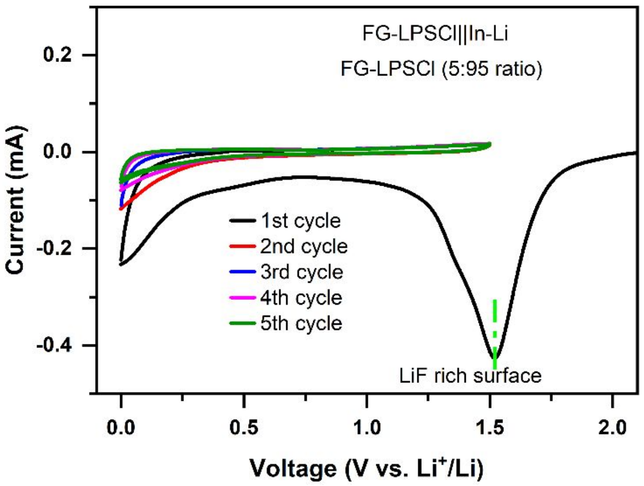

Figure S11. CV profile of the FG-LPSCl anode

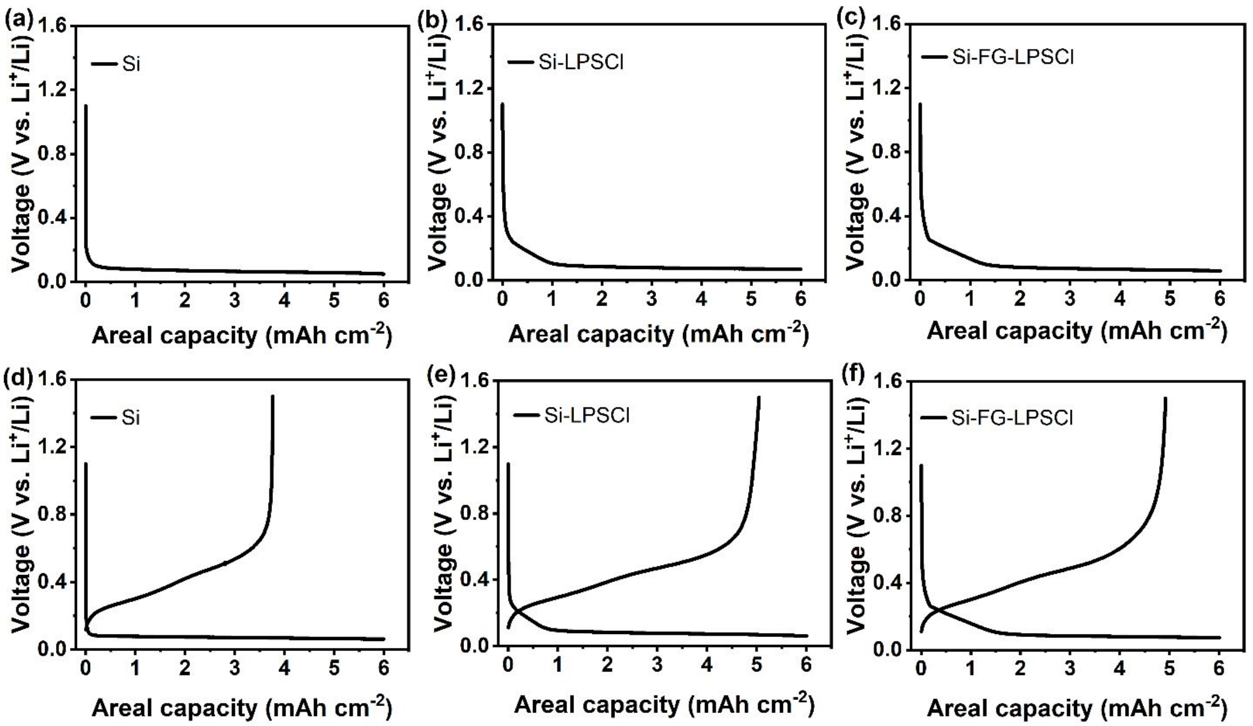

Figure S12. Lithiation and delithiation profile of Si anodes in a half-cell at a current density of 0.5 mA cm^{-2}. EIS was analyzed after first lithiation for Si (a), Si-LPSCl (b) and Si-FG-LPSCl (c) and also the EIS was evaluated after first delithiation for Si (d), Si-LPSCl (e) and Si-FG-LPSCl (f).

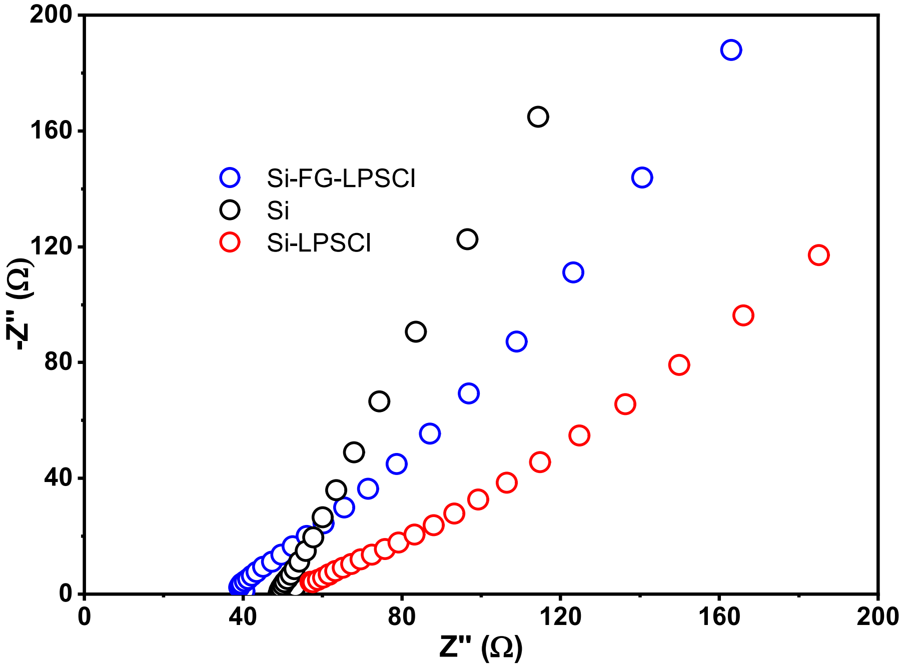

Figure S13. Nyquist plot of the three cells before cycling.

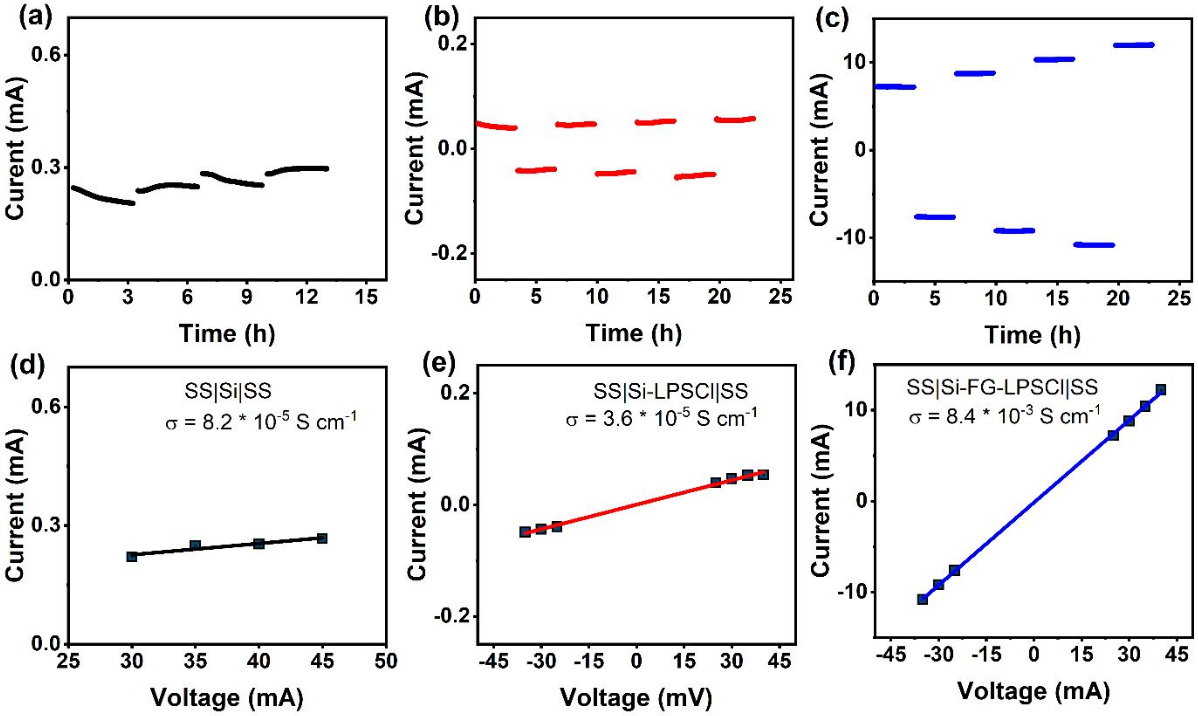

Figure S14. Ion blocking DC polarization measurements: current vs. time of Si (a), Si-LPSCl (b) and Si-FG-LPSCl (c). The corresponding linear Ohmic behavior (current vs. voltage) of Si (d), Si-LPSCl (e) and Si-FG-LPSCl (f).

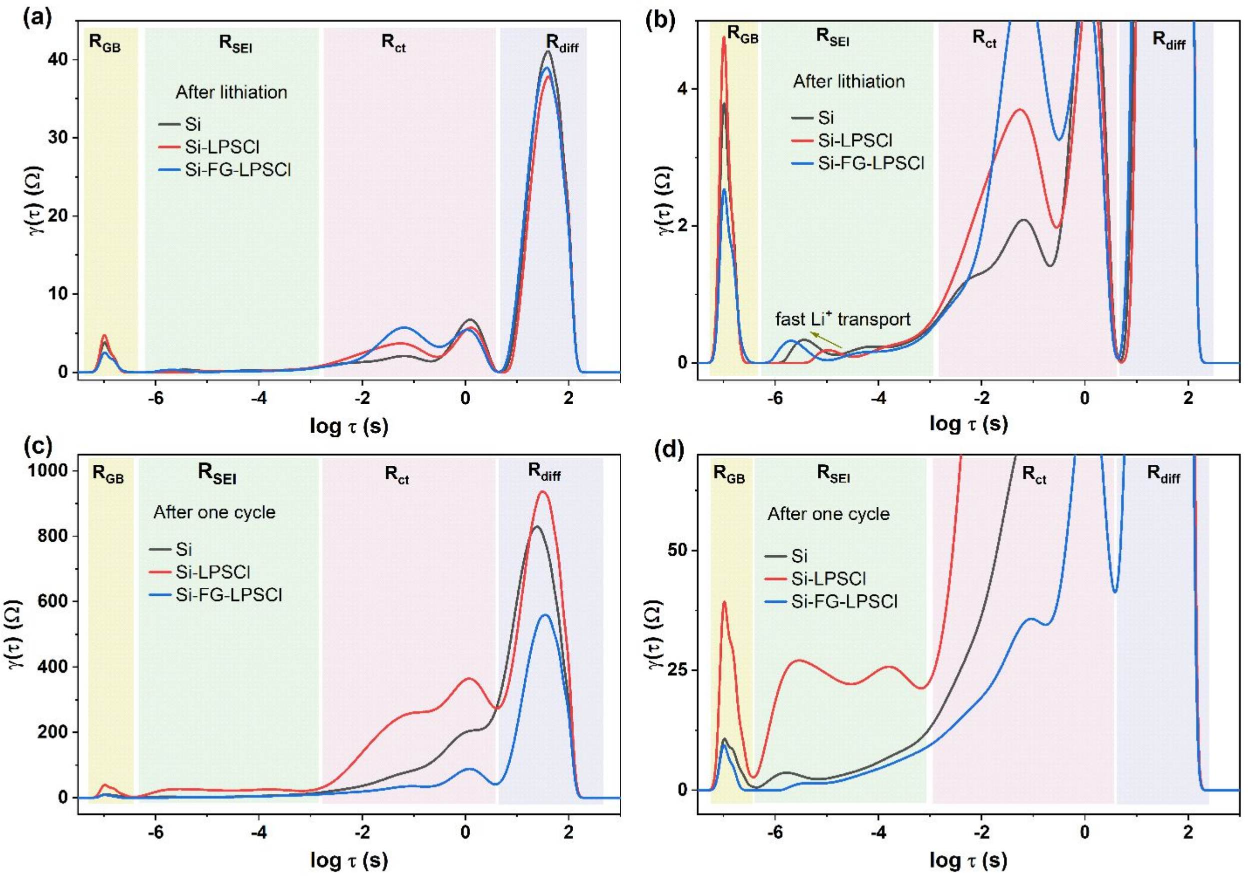

Figure S15. DRT curves of Si, Si-LPSCl, and Si-FG-LPSCl anodes during the first cycle: discharge (a) with the corresponding zoomed-in view (b); and charge (c) with the corresponding zoomed-in view (d).

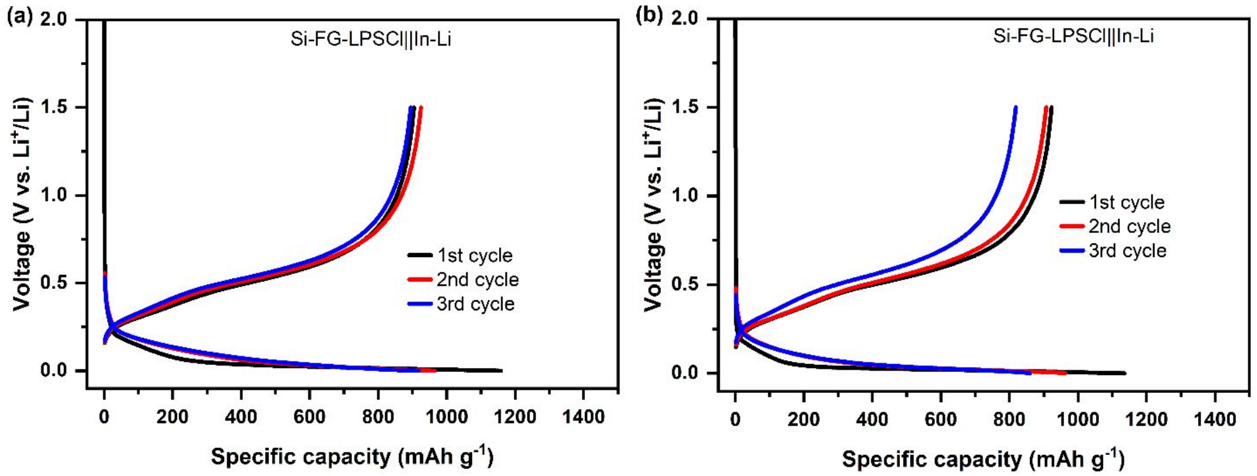

Figure S16. Charge-discharge profile of Si-FG-LPSCl composite anode with higher loadings: Si active mass loading of 4.2 mg cm^{-2} (a) and 6.3 mg cm^{-2} (b).

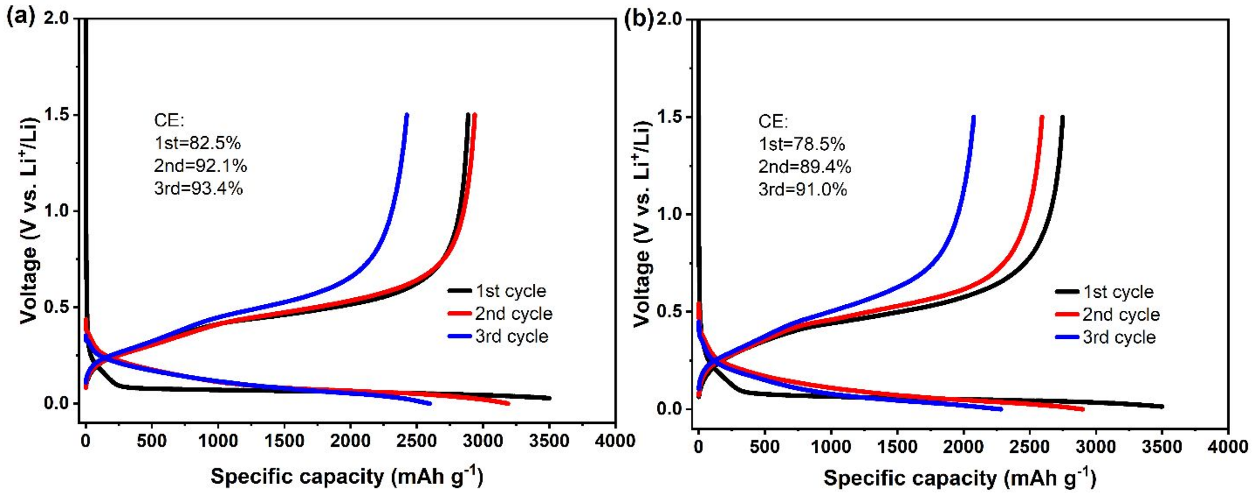

Figure S17. Charge-discharge profile of Si-FG-LPSCl composite anode with different FG loadings: Si-FG (5%)-LPSCl (a) and Si-FG (10%)-LPSCl (b).

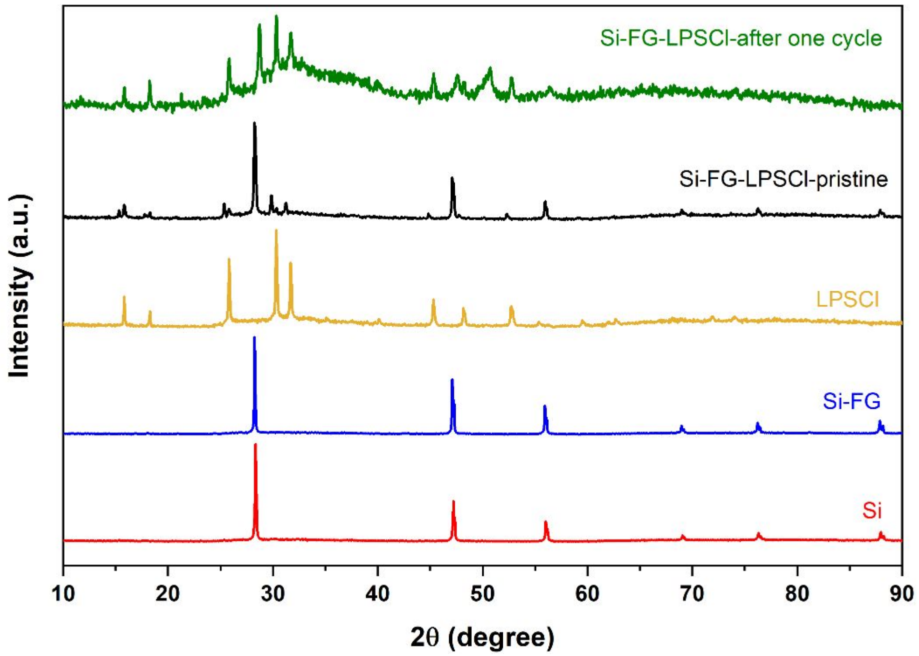

Figure S18. XRD patterns of Si, Si-FG, LPSCl, and Si-FG-LPSCl before and after cycling.

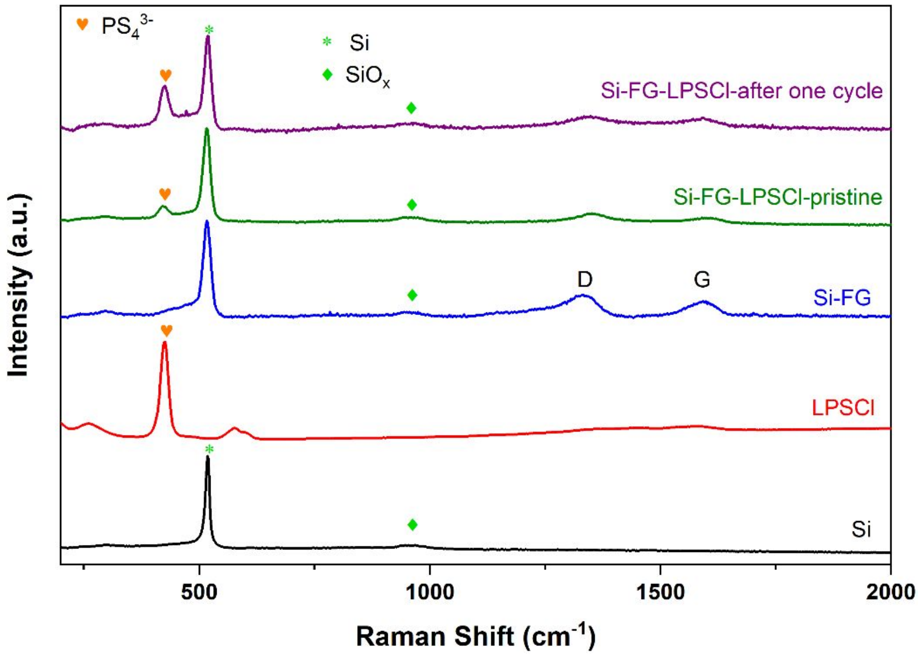

Figure S19. Raman spectra of Si, LPSCl, Si-FG, and Si-FG-LPSCl pristine and after cycling.

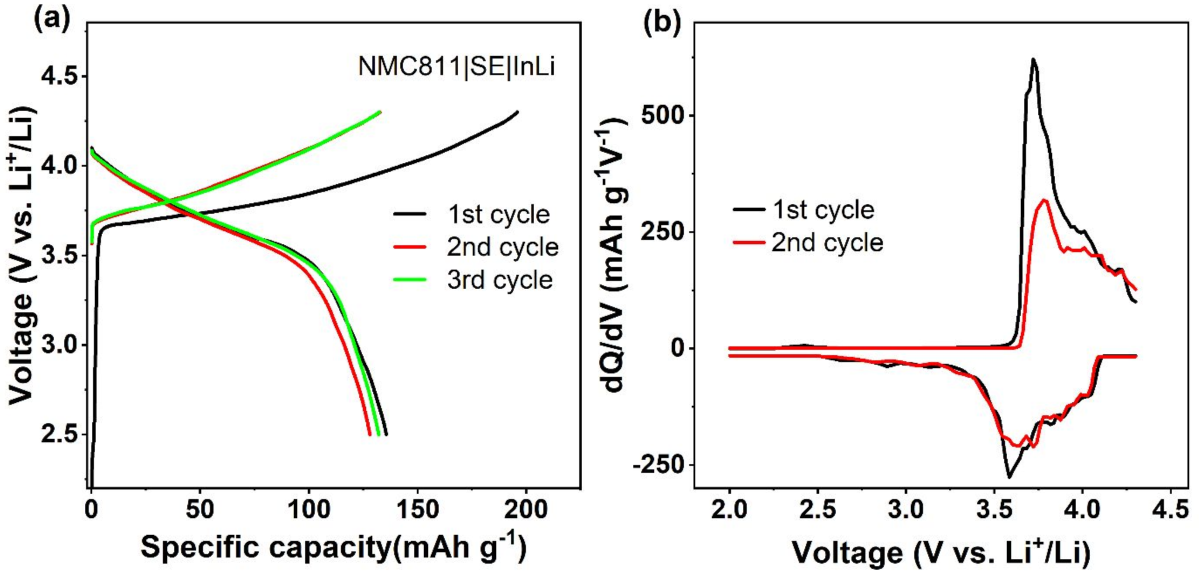

Figure S20. Half-cell profile of Nb@S-NMC811 composite cathode (a), the corresponding dQ/dV of NMC811 for two cycles (b).

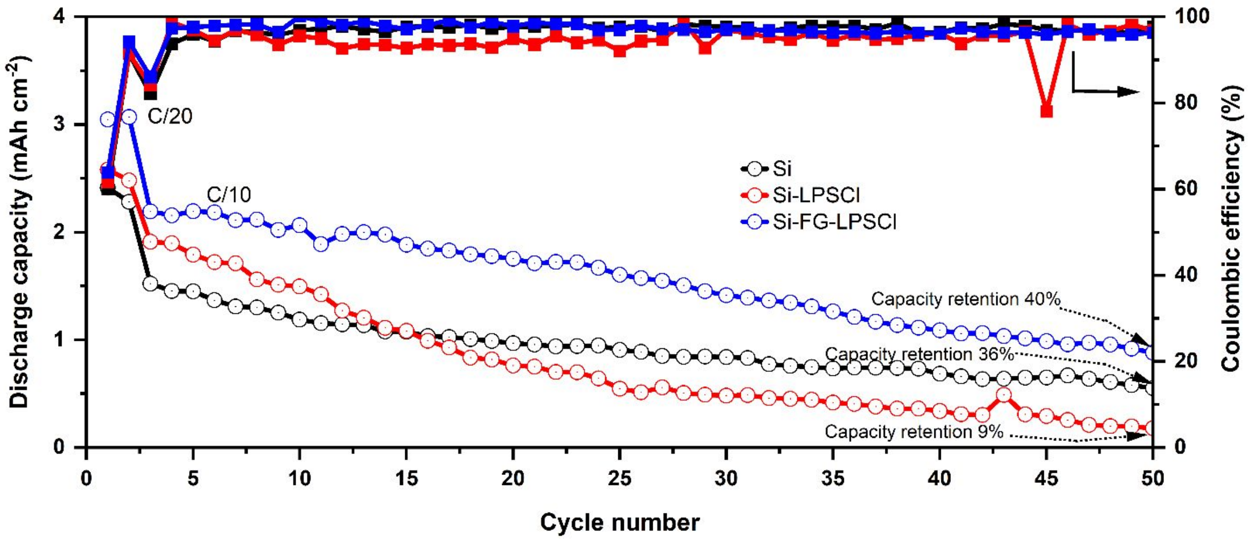

Figure S21. Cycling performance comparison by areal capacity of the three silicon anodes in a full cell with high loading of cathode, 22.93 mg cm^{-2}, the first two cycles at C/20 and then at C/10 C-rates.

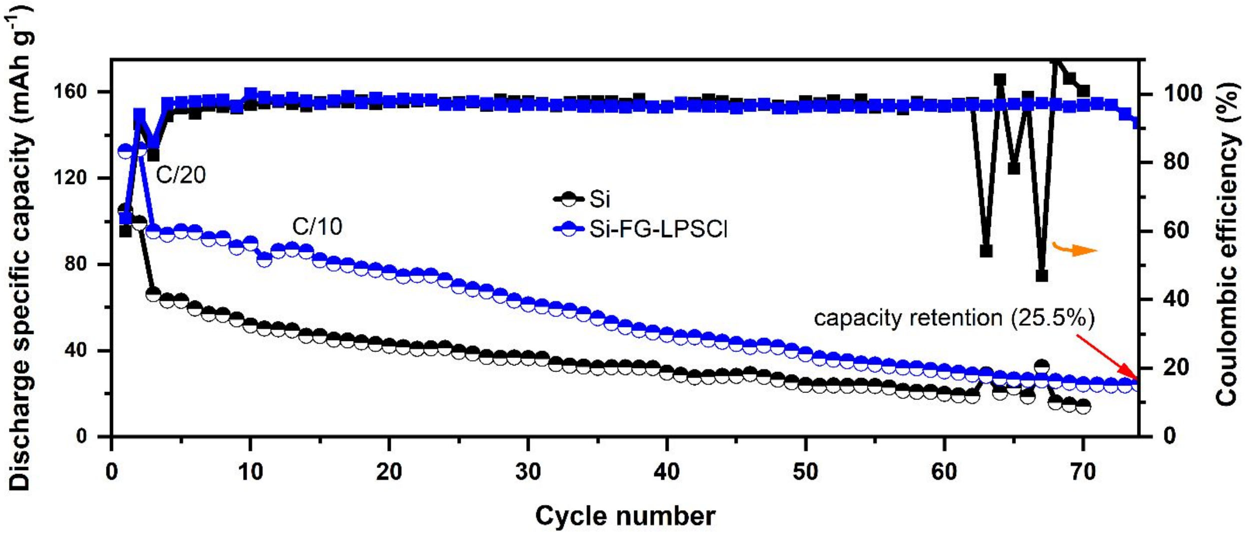

Figure S22. Full-cell comparison of Si and Si-FG-LPSCL anodes over extended cycles

Table S1. Equivalent circuit fitting results after initial lithiation of the three cells.

Elements Name	Si|SE|In-Li cell	
	Value	Fit Error
R_1	34.3950	0.7220 (2%)
P_2	0.0004	0.0001 (27%)
α_2	0.3905	0.0462 (12%)
R_2	3.2409	0.7670 (24%)
P_3	0.0193	0.0009 (4.8%)
α_3	0.5866	0.0158 (2.7%)
R_3	4.0708	0.2050 (5%)
P_4	0.0786	0.0008 (1.1%)
α_4	0.4084	0.0032 (0.79%)

Elements Name	Si-LPSCl|SE|In-Li cell	
	Value	Fit Error
R_1	30.6300	0.6110 (2%)
P_2	9.58E-06	2.24E-06 (23%)
α_2	0.5985	0.0343 (5.7%)
R_2	3.2300	0.6190 (19%)
P_3	0.0098	0.0001 (1.1%)
α_3	0.5842	0.0033 (0.56%)
R_3	14.7330	0.1650 (1.1%)
P_4	0.1236	0.0016 (1.3%)
α_4	0.4989	0.0038 (0.76%)

Elements Name	Si-FG-LPSCl|SE|In-Li cell	
	Value	Fit Error
R_1	29.5240	0.0713 (0.24%)
P_2	8.02E-05	4.69E-05 (59%)
α_2	0.6612	0.0493 (7.4%)
R_2	1.2856	0.0871 (6.8%)
P_3	0.0091	0.0001 (1.8%)
α_3	0.6440	0.0054 (0.84%
R_3	19.5230	0.3430 (1.8%)
P_4	0.1318	0.0034 (2.6%)
α_4	0.5427	0.0079 (1.5%)

Table S2. Equivalent circuit fitting results after initial delithiation of the three cells.

Elements Name	Si|SE|In-Li cell	
	Value	Fit Error
R_1	33.5110	0.1430 (0.43%)
P_2	1.09E-06	1.68E-07 (16%)
α_2	0.7621	0.0124 (1.6%)
R_2	11.1860	0.2290 (2%)
P_3	0.0012	0.0001 (10%)
α_3	0.5175	0.0217 (4.2%)
R_3	331.9300	96.5000 (29%)
P_4	0.0015	0.0001 (8.5%)
α_4	0.5004	0.0279 (5.6%)

Elements Name	Si-LPSCl|SE|In-Li cell	
	Value	Fit Error
R_1	24.6850	0.7860 (3.2%)
P_2	4.19E-05	8.80E-06 (21%)
α_2	0.4111	0.0187 (4.5%)
R_2	133.7600	18.1000 (14%)
P_3	0.0003	1.72E-05 (6.5%)
α_3	0.6844	0.0460 (6.7%)
R_3	481.1300	90.7000 (19%)
P_4	0.0009	7.54E-05 (8.3%)
α_4	0.3109	0.0151 (4.9%)

Elements Name	Si-FG-LPSCl|SE|In-Li cell	
	Value	Fit Error
R_1	38.5760	0.2080 (0.54%)
P_2	1.18E-09	4.64E-09 (39%)
α_2	0.9821	0.0284 (2.8%)
R_2	4.6149	0.2330 (5%)
P_3	0.0023	2.00E-05 (0.86%)
α_3	0.3778	0.0017 (0.46%)
R_3	185.0000	4.6200 (2.5%)
P_4	0.0063	5.00E-07 (1.3%)
α_4	0.4982	0.0032 (0.64%)

Table S3. Comparison of the electrochemical performance of recently reported works of silicon anodes in SSBs.

Si mass loading (mg cm^{-2})	Initial discharge/charge capacities (mAh g^{-1})	ICE (%)	Current density (mA cm^{-2})	References
2.10	3499/2994	85.6	0.58	This work
0.95	2773/2373	85.6	0.1	[5]
2.51	3483/2974	85.4	0.75	[6]
1.53	2111/2030	96.2	0.50	[7]
45 nm thickness	3387/2033	60.1	0.04	[8]
1	3459/2912	84	0.06	[9]

REFERENCES

(1) Chen, X.; Fan, K.; Liu, Y.; Li, Y.; Liu, X.; Feng, W.; Wang, X. Recent Advances in Fluorinated Graphene from Synthesis to Applications: Critical Review on Functional Chemistry and Structure Engineering. *Adv. Mater* **2022**, *34* (1), 2101665. DOI: https://doi.org/10.1002/adma.202101665.

(2) Jeon, K.-J.; Lee, Z.; Pollak, E.; Moreschini, L.; Bostwick, A.; Park, C.-M.; Mendelsberg, R.; Radmilovic, V.; Kostecki, R.; Richardson, T. J.; et al. Fluorographene: A Wide Bandgap Semiconductor with Ultraviolet Luminescence. *ACS Nano* **2011**, *5* (2), 1042-1046. DOI: 10.1021/nn1025274.

(3) Cheng, L.; Jandhyala, S.; Mordi, G.; Lucero, A. T.; Huang, J.; Azcatl, A.; Addou, R.; Wallace, R. M.; Colombo, L.; Kim, J. Partially Fluorinated Graphene: Structural and Electrical Characterization. *ACS Appl. Mater. Interfaces.* **2016**, *8* (7), 5002-5008. DOI: 10.1021/acsami.5b11701.

(4) Javaid, S.; Anjum, M. A. R.; Khan, R. T. A.; Akhtar, M. J. Electronic structure of partially fluorinated graphene: The impact of adsorption patterns and dynamic stability. *Chem. Phys. Lett.* **2022**, *803*, 139807. DOI: https://doi.org/10.1016/j.cplett.2022.139807.

(5) Cao, D.; Sun, X.; Li, Y.; Anderson, A.; Lu, W.; Zhu, H. Long-Cycling Sulfide-Based All-Solid-State Batteries Enabled by Electrochemo-Mechanically Stable Electrodes. *AAdv. Mater.* **2022**, *34* (24), 2200401. DOI: https://doi.org/10.1002/adma.202200401.

(6) Kim, D.-H.; Noh, S.-H.; Ha, Y.-C.; Lee, D. G.; Han, J. T.; Choi, J.-H.; Park, C.-M. Efficient Fabrication of High-Capacity Silicon Composite Anodes for All-Solid-State Lithium-Ion Batteries. *ACS Mater. Lett.* **2025**, *7* (4), 1211-1218. DOI: 10.1021/acsmaterialslett.5c00068.

(7) Huang, Y.; Shao, B.; Wang, Y.; Han, F. Solid-state silicon anode with extremely high initial coulombic efficiency. *Energy Environ. Sci.* **2023**, *16* (4), 1569-1580. DOI: 10.1039/D2EE04057C.
(8) Chen, C.; Li, Q.; Li, Y.; Cui, Z.; Guo, X.; Li, H. Sustainable Interfaces between Si Anodes and Garnet Electrolytes for Room-Temperature Solid-State Batteries. *ACS Appl. Mater. Interfaces.* **2018**, *10* (2), 2185-2190. DOI: 10.1021/acsami.7b16385.
(9) Cangaz, S.; Hippauf, F.; Reuter, F. S.; Doerfler, S.; Abendroth, T.; Althues, H.; Kaskel, S. Enabling High-Energy Solid-State Batteries with Stable Anode Interphase by the Use of Columnar Silicon Anodes. *Adv. Energy Mater.* **2020**, *10* (34), 2001320.
